# Tracing early life stress in human molar morphology: Associations between linear enamel hypoplasia and maxillary first molar form

**DOI:** 10.1371/journal.pone.0354698

**Published:** 2026-07-29

**Authors:** Erin C. Blankenship-Sefczek, Alan H. Goodman, Mark Hubbe, John P. Hunter, Timothy M. Sefczek, Debbie Guatelli-Steinberg

**Affiliations:** 1 Department of Oral Biology, School of Dentistry, Creighton University, Omaha, Nebraska, United States of America; 2 School of Natural Sciences, Hampshire College, Amherst, Massachusetts, United States of America; 3 Department of Anthropology, University of Tennessee, Knoxville, Tennessee, United States of America; 4 Department of Evolution, Ecology and Organismal Biology, The Ohio State University, Newark, Ohio, United States of America; 5 School of Global Integrative Studies, University of Nebraska, Lincoln, Nebraska, United States of America; 6 Department of Anthropology, Ohio State University, Columbus, Ohio, United States of America; Tribhuvan University, NEPAL

## Abstract

Permanent tooth structures can respond to environmental disruptions even after the initiation of hard tissue deposition at the cusp tips, affecting dental morphology. In bioarcheological studies, linear enamel hypoplasia (LEH) provides evidence of both the presence and timing of developmental stressors that can occur during first molar odontogenesis. Yet, previous studies used LEH from the entirety of the anterior tooth surface to relate disruptions to changes in permanent tooth morphology, which could conflate causality by misrepresenting the timing of events. This study assessed associations between LEH formed during maxillary permanent first molar development and dental characteristics of these molars in contemporary humans with known health and nutritional histories from Tezonteopan, Mexico. LEH were observed in the field, and dental characteristics (crown size, principal cusp spacing, expression of cusp 5 and Carabelli trait) were observed on maxillary dental casts taken of the same adolescent study participants (n = 54). Statistical analyses included multivariate- and generalized linear models, and Principal Component Analyses. Slightly larger crown sizes, decreased buccolingual principal cusp spacing, and a slight decrease in cusp 5 expression were associated with the presence of LEH. The decrease in cusp spacing and decrease in cusp 5 expression follow expectations of the Patterning Cascade Model. Our results differ in some ways from those of previous studies possibly due to our focus on LEH defects forming during molar developmental periods. Our findings support the growing body of studies highlighting the unpredictability of molar morphology in the presence of developmental disruptions.

## Introduction

There is a long-held assumption suggesting that dental developmental processes (e.g., crown size determination, cusp presence, eruption timing) demonstrate high heritability [1–3]. However, associations between developmental disruptions and altered permanent tooth characteristics have been identified in studies assessing populations with known health histories as well as ones that approximate stress via dental markers (i.e., enamel hypoplastic defects). Altered dental characteristics include changes to tooth crown size [4,5], principal cusp spacing [6], and accessory trait expression [7,8]. Indeed, Skinner and colleagues [9], based on patterns from previous studies, comment on a noticeable decrease in heritability of tooth size over the last few decades and argue that environmental factors explain nearly one-third of the variation observed in the dental phenotype. These trends highlight the unpredictable outcomes to dental morphology in times of developmental disruptions.

Permanent dental morphology is resultant from intricate signaling mechanisms during the developmental process. Perturbations during the developmental period have been associated with changes to the onset timing of tooth formation stages, with some stages also experiencing prolonged growth periods [10,11], and altered tissue responses or activity at nearly every stage of odontogenesis [12–14]. For example, nutritional deprivation is known to influence hormone secretion, which in turn influences protein formation associated with tooth germ tissue activity [15,16]. The outcomes appear highly variable with crown size either increasing or decreasing [12,17], overall crown height and root length increasing or decreasing [13], timing of tooth formation stages delayed or remaining unchanged [10,11], eruption timing accelerated or delayed [18], and molar cusps larger, smaller, fused or missing [7,19,20]. The unpredictable nature of this variation may be largely due to a lack of correlating the timing of perturbation events with dental development. A disruption occurring while first molar morphology is determined may directly influence this process, whereas a perturbation experienced after that period might affect root development or the formation of supporting dental tissues. Therefore, timing is an important factor when assessing how environmental disruptions might affect dental developmental processes associated with molar morphology.

Our study represents a unique opportunity to focus on associations between timing of external stress events and morphological outcomes to permanent molar structures in a group of individuals with enamel defect data and known health information. We use linear enamel hypoplasia (LEH) in this study because many bioarchaeological projects rely solely on enamel defects to approximate developmental stress events [21]. However, links between LEH and dental characteristics show mixed results [e.g., 4–5,7], possibly due to timing differences, as detailed below. To explore the relationship between defect timing and dental characteristics, an understanding of timing of molar developmental events and anterior tooth enamel formation is important for evaluating possible associations between ontogenetic markers (LEH) and change in molar tooth size and shape. Therefore, in this introduction we provide: (1) an overview of odontogenetic events, (2) a review of what is known about upper first molar cusp development timing, (3) a discussion of the predictors of molar shape, (4) a review of the calcification of anterior teeth and its timing, and (5) the hypotheses and predictions guiding this study.

### Events of odontogenesis

Tooth development occurs through reiterative signaling, which regulates cell activity in the dental epithelium and underlying neural-crest-derived ectomesenchyme at various stages of odontogenesis [22,23]. The first sign of a developing tooth is a localized thickening of epithelium at the site of that future tooth. Specific tooth morphology is first identifiable in the early bell stage, where the definition of the occlusal surface with some distinct cusp tips and marginal ridges appears due to extensive epithelial downfolding [24]. In upper molars, the mesiobuccal cusp (paracone) appears first, followed by the mesiolingual cusp (protocone), then the distobuccal cusp (metacone) and lastly the distolingual cusp (hypocone). Although principal cusp morphology begins to be visible by the early bell stage, the actual spacing of principal cusps is not fixed until the valleys between cusps are completely bridged with mature (fully mineralized) enamel [25,26]. This occurs later in the crown stage as, for multi-cusped teeth, the valleys between cusps are the last regions where enamel deposition occurs.

Early in odontogenesis, a transient cluster of non-dividing cells called the primary enamel knot appears to direct epithelial downfolding associated with morphogenesis (tooth shape) determination [27,28]. In multi-cusped teeth, secondary enamel knots form within the epithelium at the tip of each future cusp during the later bell stage [29]. Each enamel knot, primary or secondary, emits growth-activating and growth-inhibiting signals that regulate where cell activity can occur and where it cannot [27,30]. Cell proliferation associated with cusp development can only occur in regions of the occlusal surface where inhibitory signals are attenuated [19,27,31]. In this way, for multi-cusped teeth, the first cusp to develop regulates the presence and spacing of later-forming cusps [22].

### The known and unknown about upper first molar cusp development timing

All human teeth, regardless of class and corresponding shape, follow the stages described above. What differs among tooth types is initiation timing, growth rate, and duration of growth or timing of termination signaling [24,25,32–35]. Tooth types displaying multiple cusps, such as molars, have more complex development because cusp formation is regulated by the cusps that initiate earlier [22]. Furthermore, as discussed below, human upper molar mesial and distal cusp pairs demonstrate allometric growth patterns, meaning the timing, rate, and quantity of mitotic activity associated with the soft tissue of each cusp or cusp pair is slightly different [24,25,36,37]). The result of this allometric, or differential, growth is the possibility for varied principal cusp spacing configurations [38].

**Fig 1:**
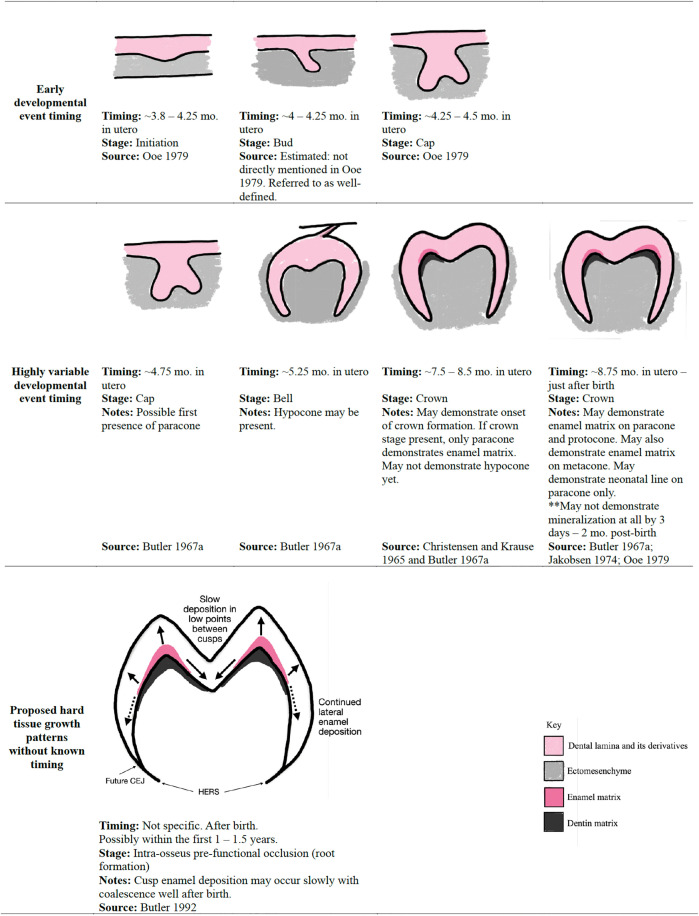
Molar developmental events and variation in timing estimations. Note: for the last section titled “Proposed hard tissue growth patterns without known timing”, the image depicts a tooth that has entered root formation (shown by the presence of Hertwig’s epithelial root sheath (HERS) and future site of the cemento-enamel junction (CEJ)) but has not completed hard tissue deposition. The solid arrows represent the direction of enamel matrix secretion by secretory ameloblasts. The dotted arrows represent the regions where ameloblast differentiation will occur as hard tissue deposition continues along the lateral regions of the crown.

Evidence of early fetal growth suggests there may be general trends in the timing of odontogenetic stages but that considerable variation exists for the onset of these stages and for the appearance of specific cusps [24,33,34] (see Fig 1 for a summary of events). Ooe [34] found that upper first molars may initiate around 3.8 months (110 mm crown-rump length) and enter the cap stage around 4 months (140 mm crown-rump length) in utero. Butler [24] noted that around 4.75 months in utero (19–20 weeks) the paracone may be first identifiable. By around 5.25–6 months gestation (21–24 weeks) the molar may enter the early bell stage and some individuals may show the first presence of the distolingual cusp (hypocone) [24]. Butler [24] described some teeth at this age as showing more definition in cusp shape but without hard tissue secretion, suggesting the transition to the early bell stage by this time.

Christensen and Kraus [33] indicated that the average age associated with transitioning to the crown stage may be around 30 weeks or 7.5 months in utero. However, they also cautioned that considerable variation in calcification timing of upper molar cusps exists because not all cusps they observed showed signs of hard tissue formation before birth [33]. The earliest calcification detected was around 7 months in utero (28 weeks) and was associated with just the paracone. The oldest age at which they found calcification occurring in at least one cusp, also the paracone, was around 8 months in utero (32 weeks) [33]. Similarly, Butler [24] found that around 7.5–8.5 months gestation (30–34 weeks), only some individuals showed evidence of paracone calcification, and one individual had not yet manifested initiation of a hypocone. By 8.75 months in utero (35 weeks), Butler [24] noted that some, but not all, individuals showed calcification confined to the tip of the protocone and the appearance of a rhomboid occlusal shape. These findings suggest that by 35 weeks gestation, the crown stage was reached in some, but not all individuals observed by Butler [24]. Adding to this variation, Ooe [34] found that some individuals showed no mineralization of any cusp three days to two months post-birth. This timing is supported by Reid and Dean [39] who indicated that initiation of enamel formation for the paracone and protocone occurs around the time of birth. Near the end of gestation (roughly 36–40 weeks), Butler [24] describes cuspal development of the paracone and protocone as being slow in the occlusal regions where the cusps eventually bridge. For an individual at term or just after birth (about 36–42 weeks), Butler [24] described the occlusal regions as showing the least amount of calcification compared to the margins of the cusps and noted that distances between cusps showed the highest growth rates. The oldest individual in Butler’s [24] study aged around 42 weeks, showed minimal amounts of calcification confined to the tip of the protocone and metacone. Similarly, Jakobsen [40] found that typically the paracone was the only cusp developed enough to demonstrate a neonatal line.

After cusps initiate during the soft tissue period of tooth formation, distances between them increase, leading to cuspal spreading throughout odontogenesis [25,41,42]. At first, this spreading may be due to continued epithelial growth at each cusp basin [25]. As the process continues, cusps appear to spread to a disproportionally greater degree than base enlargement, and mesial cusps appear to spread more slowly than distal cusps [25]. Later in development after calcification has begun, this process of cuspal spreading appears to continue until enamel matrix deposition converges in the regions between cusps [25]. This continued spreading may occur because as ameloblasts move away from the enamel-dentin junction, cusp tips on the enamel surface migrate either lingually or buccally relative to the underlying dentin horns [41]. The outer enamel surface also shows greater shape variability compared to the enamel-dentin junction, suggesting that differences in amelogenesis may contribute to both tooth shape and covariation between teeth [42].

It is well known that mineralization of upper first molars starts around the time of birth and finishes between 30–48 months post-natal [39,43,44]. What remains unclear is the specific timing of enamel convergence within the valleys between upper first molar cusps. Kono and colleagues [45] found that the protocone and hypocone show the greatest enamel thickness. Enamel thickness has been found to have a positive correlation with the duration of active secretory ameloblasts where a longer secretory phase results in prolonged growth periods [46]. In addition to thick cuspal enamel, molar occlusal surfaces demonstrate an increase in topography (i.e., the high and low points associated with cusps, crests, and ridges) compared to the marginal surfaces [47,48] and many ridges show relatively thicker enamel [45]. These findings would suggest that regions of the upper molar occlusal surface may have a prolonged period of active secretory ameloblasts before enamel convergence takes place. In accordance with these observations, Reid and Dean [39] found variation between populations in the timing of paracone and protocone growth rates, although they did not specify times of cusp coalesce. Furthermore, Butler [26] suggests that the fixation of permanent upper molar cusp spacing does not occur until well after birth.

### Predicting molar trait expression

Molar occlusal morphology is dependent on the result of allometric growth patterns [24,25,36,37]. Variation in cusp size and spacing can be predicted by the Patterning Cascade Model (PCM) [22] of tooth development. Specifically, the PCM proposes that later-forming cusps are related to crown size, a proxy for the duration and rate of tissue growth, as well as cusp spacing, a proxy for the size of inhibitory zones surrounding cusps [8,22,49–53]. In testing the PCM, researchers have approximated dimensions of crown size relative to cusp spacing using 2D [8,22,51–53] and 3D measurement techniques [54,55]. From these studies, specific configurations of upper molar principal cusps have been predicted to influence the formation and size of any accessory cusps, such as upper Carabelli trait or upper cusp 5, that initiate after the four upper principal cusps [8,51,55]. For example, on the same size crown, smaller buccolingual dimensions between the paracone and protocone may create an optimal environment for Carabelli trait expression [51]. Similarly, reduced mesiodistal dimensions of all four principal cusps may provide an optimal environment for the expression of Carabelli trait [8]. Therefore, molar morphogenesis occurs in a developmental cascade wherein crown size and principal cusp spacing configuration influences the presence and size of any later-forming cusps [22]. However, as already indicated, relationships between crown size and environmental perturbations are variable, as molar crown size may increase with maternal hypothyroidism and diabetes [17] or in the presence of third molar agenesis [12], while size can decrease in times of severe malnutrition [56–59]. Therefore, changes can create inconsistencies in the dental characteristics associated with PCM predictions.

### Timing of upper anterior tooth calcification

While first permanent molars undergo many developmental stages during gestation, permanent anterior teeth initiate and complete crown growth after birth. Permanent upper central incisors begin enamel formation around 3–5 months after birth, lateral incisors between 6–10 months after birth, and canines by around 7–10 months after birth [39,60]. Crown growth then occurs from cusp tip at the enamel-dentin junction, to the tooth cervix with differing formation rates along the crown [39]. For all teeth, the cuspal or incisal region corresponds to earlier periods of enamel formation and development while regions closer to the cervical margin correspond with later periods of enamel formation [39,61]. For example, around one year, permanent upper central incisors show about 10% crown formation, by about 2.2 or 2.4 years 50% is complete and by about 4.2 or 5.0 years crown formation is 100% complete [39]. Permanent upper canines show similar timings, with about 10% crown completion at around 1.9 years, 50% completion around 2.8 or 3.0 years and 100% completion around 4.8 or 5.3 years [39].

Based on our understanding of anterior tooth enamel formation rates and the post-birth period of molar cusp development, it seems possible that some overlap in timing between these two processes may occur. For example, it seems plausible that enamel convergence in the valleys between cusps would occur at least by the time about 60% of the upper first molar crown is formed, which occurs around 1.9 or 2.1 years after birth [39] By this time, the upper central incisors have formed roughly 40% of the crown, and upper canines about 10% of the crown [39]. In both cases, enamel formation of the upper central incisor and upper canine would be past the cuspal region and have moved to lateral enamel formation. This would suggest that some enamel hypoplasia in anterior teeth, particularly in the earlier periods of enamel formation, may overlap with the final determination of molar principal cusp spacing. Furthermore, because growth continues along the margins of the molar crown after cusp spacing is set [26], later-forming enamel hypoplasia in anterior teeth may overlap in timing with molar crown growth.

These specific timing overlaps may explain why studies using enamel defects on anterior teeth have shown inconsistent associations between developmental disruption and dental characteristics [4–8]. We consider two possible pathways that could explain this seemingly variable relationship. One is that LEH defects on anterior teeth represent periods of developmental disruptions that do not coincide with the periods during which crown size and cusp spacing are completed. For example, an LEH formed in the 4^th^ year after birth would not represent a stress event that occurred during first permanent molar development. This line of reasoning predicts no association between LEH presence and molar variation. The other possibility is that an individual who is stressed once is more likely to be stressed again or at least be more depleted and susceptible to another stress. In this way, there may still be associations between developmental events that do not directly overlap in time, for example, between events of molar morphogenesis and later events producing LEH in anterior teeth.

### Hypotheses and predictions

In a previous study [8], we demonstrated a relationship between nutritional status and changes to molar characteristics in two groups from Tezonteopan, Mexico: one that received nutritional supplementation from gestation through adolescence and one that did not [62]. The two samples differed with respect to their nutritional status as members of the supplemented group had access to extra food that allowed their nutrition intake to more closely align with recommended daily caloric and protein recommendations (1,250 calories and 32g protein per day), whereas the non-supplemented group consumed only the traditional diet that was well below the recommended average for nutritional intake (roughly 725 calories and 19.2g of protein per day) [62]. We found that various aspects of the non-supplemented group’s molar features, such as reduced mesiodistal principal cusp spacing as measured by cusp tips on the enamel surface, and an increase in accessory trait expression, differed from the supplemented group’s, a likely consequence of these prolonged nutritional stress in the non-supplemented group [8]. At the time of the original study, Goodman and colleagues [63] found that linear enamel hypoplasia (LEH) was more prevalent in the non-supplemented group. Given that many bioarchaeological studies rely on enamel defects as indicators of developmental disruptions, and that timing of disruptions is an important predictive factor, for the present study we focus on how these elements may be associated with first molar morphology.

Here, we build on our previous research by assessing the relationship between the presence of linear enamel hypoplasia on the maxillary anterior dentition (central incisors, lateral incisors, canines) within specific developmental zones and changes to maxillary first molar dental characteristics (crown size, principal cusp spacing, accessory trait expression) in a sample of contemporary adolescents (n = 54) from Tezonteopan, Mexico, with known nutritional intake from gestation to adolescence [62]. We hypothesize that the occurrence of LEH is associated with changes to some molar characteristics, with specific predictions based on findings from our previous study [8]. Our baseline assumption is that the molar characteristics that we found in the nutritionally compromised group would be similar to those we would find in individuals with LEH defects that formed during the period of first molar development. Thus, for first molar crown size we predict no change in size when LEH is present, consistent with our previous work and contra findings of McKee and Lunz [4] who found a decrease in molar size in individuals with LEH present. For principal cusp dimensions, we predict that with the presence of LEH, there will be a reduction in mesiodistal cusp spacing, as there was in the nutritionally compromised sample. Likewise, for accessory trait expression we predict an increase in the presence of upper cusp 5 and Carabelli trait when LEH is present. Lastly, we predict that accessory trait expression will be related to distances among cusps relative to crown size, as expected on the basis of the PCM [8,51,54,55].

## Materials and methods

Beginning in 1968, Dr. Adolfo Chavez and nutritionist Celia Martinez conducted a longitudinal study on the effects of nutritional intake on growth and development in the rural Mexican town of Tezonteopan [62]. During this time-frame, about 80 subadults were monitored from infancy through adolescence and information was collected about dietary habits, height, weight, and body mass index [62]. Roughly half of the study participants were enrolled in a governmental program called Proyecto Puebla which provided dietary supplementation to qualifying families in the rural community. Women enrolled in this program were provided supplements at the first sign of pregnancy, and their children continued to receive these after birth [62]. Overall, Chavez and Martinez [62] found that individuals in Tezonteopan had a poor base diet, with most classified as mild or moderately malnourished, and experienced bouts of infection.

In an associated study, Alan Goodman recorded enamel defect type and location directly from anterior dentition of study participants [63]. This portion of the study was approved by the Mexican National Institute of Nutrition and Hampshire College’s Institutional Review Board (IRB) [63]. Informed written consent was obtained prior to participation in the study. After enamel defects were recorded, Goodman and colleagues [63] made dental impressions of the maxillary arches from all study participants. For the present study, principal cusp spacing and accessory cusp expression data were collected from these casts, not from living human subjects. In 2015, an IRB application was submitted through Buck-IRB at Ohio State University’s (OSU) Office of Responsible Research Practices. Because the materials for tooth measurement and recording were taken from casts rather than from living subjects, OSU’s Buck-IRB determined that IRB approval was not required for this portion of the study.

Dentitions were measured from casts, housed in the School of Natural Sciences at Hampshire College, of subadults and young adults with ages ranging from 10 to 20 years. Permanent first maxillary molars have been identified as a key tooth for observing some accessory traits [64]. For this reason, casts with at least one permanent molar and less that 5% estimated occlusal wear were used for data collection. Based on these criteria and presence of enamel defects, 54 individuals were included in this study. Observations of crown size, principal cusp spacing, and cusp expression were undertaken by EBS who was blinded to participant information during data recording. Measurements were taken from casts rather than 2D or 3D images because previous studies have found no statistically significant differences in dental metrics between these modalities [65,66]. Recording enamel defects was undertaken by AHG who was also blinded to participant information at the time of data recording.

### Enamel defects

Enamel defects were recorded directly from dentition of the study participants, not from casts, during routine dental cleanings in June of 1988 by AHG. Defects were recorded by location and type on the labial surface of all maxillary anterior teeth (left and right central and lateral incisors, and canines) following the epidemiological standards for classification of developmental defects of dental enamel (DEE index) of the Federation Dentaire International [63,67]. Five types of defects were found – three types of opacities and two types of quantitative defects [63]. Given the ubiquitous use of LEH in anthropological research, we used defects classified as horizonal-line or grove hypoplasia (also referred to as linear enamel hypoplasia) [63] for analysis in the current study. Intraobserver reliability was found to be high (88.9% agreement) for recording of defect type and location.

**Fig 2:**
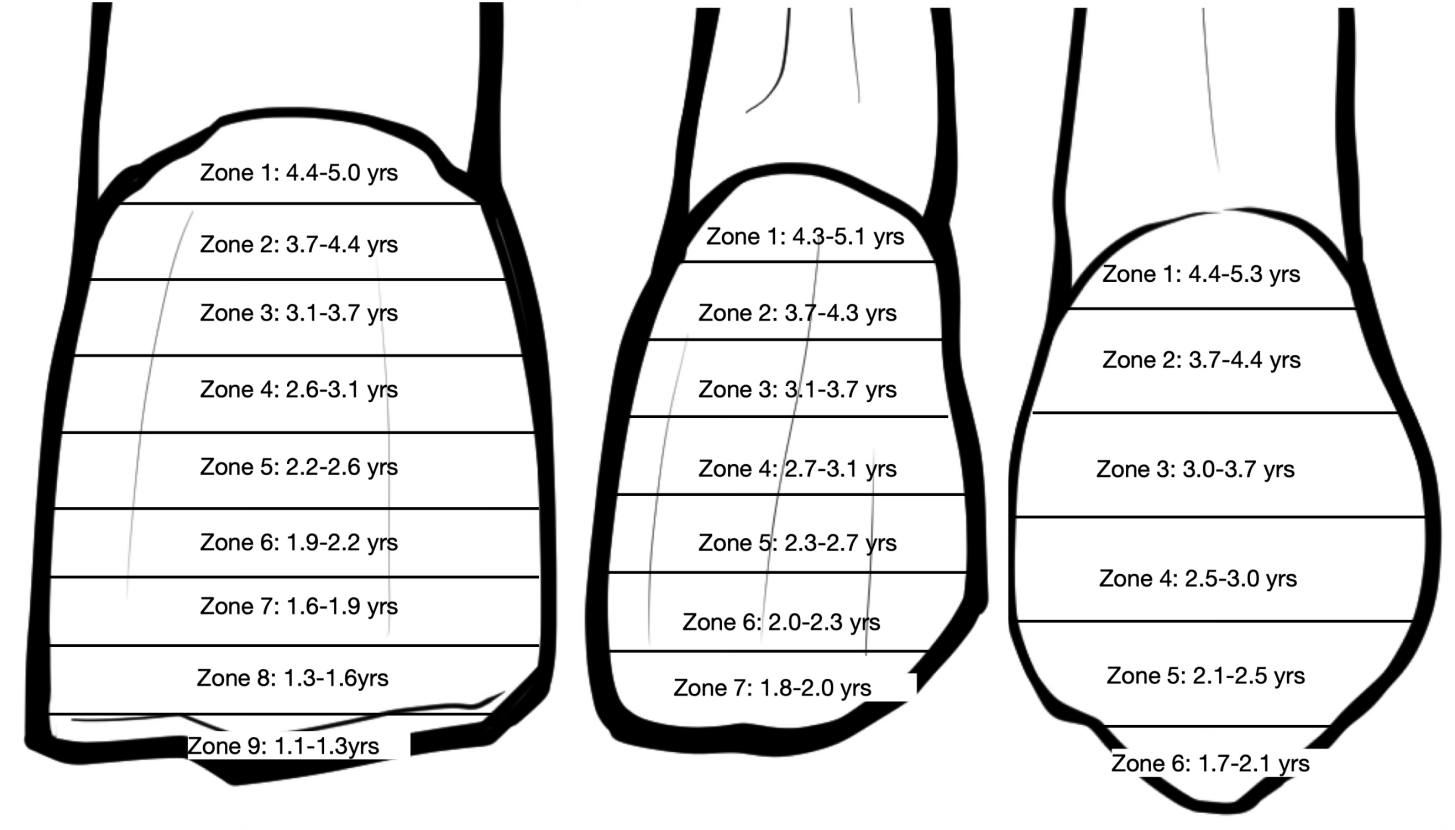
Estimated enamel formation times of zones for upper anterior dentition. Zone age ranges were calculated using Reid and Dean’s [39] estimations and Hillson’s [70] visualizations.

To better match defect location to age of onset, incisors and canines were divided into zones that corresponded with a timeframe of development. Following standards outlined by Massler and colleagues [68], Goodman and colleagues [63] divided central incisors into nine zones, lateral incisors into seven zones, and canines into six zones. For the present study, age ranges for these tooth zones were updated to follow Reid and Dean’s [63] estimations of enamel formation timing. We calculated the percent of the crown corresponding with the number of zones (e.g., 16.6% per zone for upper canines). We then applied a linear interpolation to estimate age-ranges associated with each zone (Fig 2) as this method does not yield different estimations compared to nonlinear interpolations [69] To assess the possibility of using LEH as a proxy for developmental events associated with changes to molar characteristics, we only compared zones that corresponded to an overlapping period of molar occlusal morphology. Therefore, zones 9–5 on the central incisors, zones 7–5 on lateral incisors, and zones 5–6 on canines were included in statistical analyses.

We compared all dental characteristics to zones from left and right anterior dentition separately in statistical analyses. It has been suggested that matching LEH on antimeric pairs indicates the presence of systemic stress during development [60] and that unilateral, or unmatched, LEH may represent localized disruptions such as trauma [21]. However, many of the individuals included in this study demonstrated prolonged mild to moderate malnutrition from gestation through adolescence [62], which likely accounts for much of the LEH expressed in this group [63] Furthermore, antimeric pairs of permanent dentition have been shown to have different daily secretion rates, which may indicate that antimeres may not be laying down enamel in lockstep [71]. It has also been suggested that different teeth show gradients in susceptibility to amelogenesis disruptions [72], which may mean that even when development occurs contemporaneously between antimeres, each tooth may not demonstrate equal exposure or similar responses [21,71,73].

In this study we analyzed defect prevalence and number. Anthropologists have suggested that more might be gleaned about growth disruptions by measuring the depth of enamel defects, assuming that these reflect the severity of the stressor that produced them [74–77]. However, experimental studies on sheep do not support this possibility [78]. In Suckling and Thurley’s work, the dimensions of enamel defects of sheep infected with nematodes did not correspond to their parasite load. We also note that the depth of defects is affected by the angles that striae of Retzius make to enamel surfaces, and these angles vary along the length of the crown [79]. For these reasons, we did not attempt to assess defects as more or less severe on the basis of their depth.

### Molar crown size and principal cusp distances

**Fig 3:**
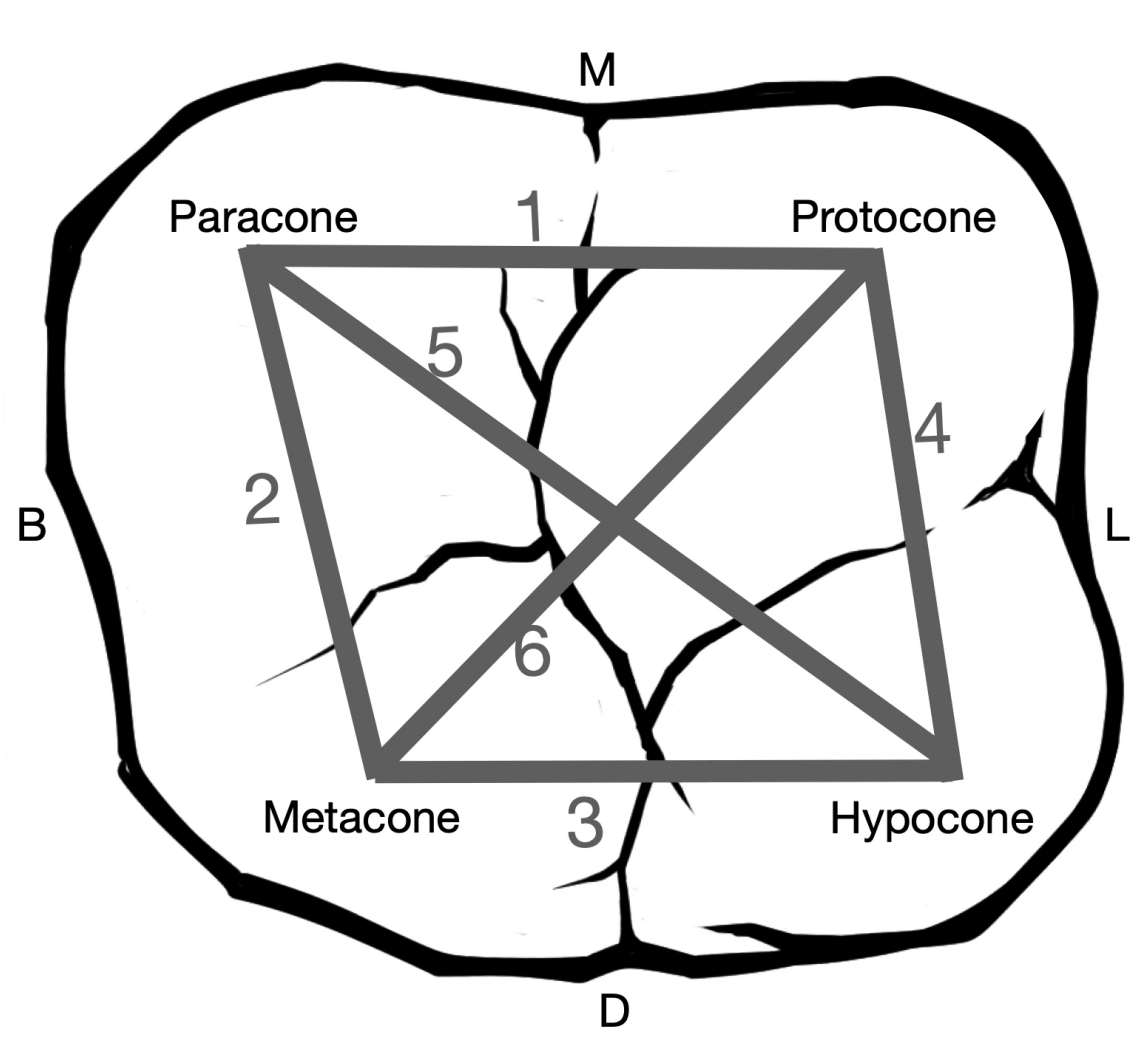
Intercusp distances of enamel cusp tips measured and compared for upper first molars. Line numbers represent dimensions between principal cusps. 1: paracone-protocone; 2: paracone-metacone; 3: metacone-hypocone; 4: protocone-hypocone; 5: paracone-hypocone; 6: metacone-protocone.

Maximum buccolingual (BL) and mesiodistal (MD) distances were taken using digital calipers [80] for right maxillary first molars [81]. Each measurement was taken three times, and the means were used for statistical analysis. To estimate crown area, BL and MD distances were multiplied (BLxMD). For principal cusp spacing, each principal cusp tip was marked and then six intercusp distances (ICDs) were measured on each tooth (Fig 3). Each distance was measured three times and the average of these measurements used for statistical analysis. Relative cusp distances (those corrected for crown size) were calculated from the absolute distance divided by the square root area of the corresponding molar area [51,53,55] and used for statistical analyses.

### Molar accessory traits

Written descriptions and associated scoring plaques from the ASUDAS [64] were used to record presence and size of first molar accessory traits. Recording for maxillary first molar Carabelli trait and cusp 5 were based on a grade of expression starting with “0” as absent and increasing as the trait expression became larger. Carabelli trait was scored from 0–7, and cusp 5 was scored from 0–5. For statistical analyses, trait frequency (presence/absence) was compared. Breakpoints were used to determine trait “presence”. Because Indigenous Central and South American populations demonstrate lower global averages of molar accessory cusps [82], breakpoints for Carabelli and cusp 5 were set at ≥ 1. Molar accessory trait data was recorded by EBS. Intraobserver reliability for dental trait scores was tested on a sub-sample of 30 individuals. Results of the One-way Interclass Correlation (ICC) statistic (0.87) showed intraobserver reliability was “good” (values between 0.75 and 0.90) [83].

### Statistical tests

We ran a series of linear models (LM), multivariate linear model (MLM), and generalized linear models (GLM) to evaluate the associations between LEH presence and dental characteristics. All statistical tests were run in version 4.4.2 of R [84] with the lme4 package [85] and brms package [86].

Development of molar crown size and principal cusp spacing are part of processes associated with reaching functional occlusion. Although there is likely some integrated development between these variables, each could also be the result of independent factors that ultimately lead to the final expression [87]. Furthermore, we found small effect sizes (S1 Table) for an association between crown size and cusp spacing. For these reasons, we analyzed molar crown size as a separate variable from cusp spacing. For intercusp distances, we calculated Principal Component (PC) scores for the six principal cusp distances (Table 1) to address multicollinearity among these response variables. Only the first three PC scores were used in LMs, MLMs and GLMs to address the influence of developmental defects on dental characteristics.

**Table 1:** Principal Component analyses for upper first molar principal cusp spacing.

	PC1	PC2	PC3
SD	1.658	1.054	0.973
Proportion of variance	0.458	0.185	0.158
Cumulative proportion	0.458	0.643	0.801
PC weights by variable			
ICD 1	0.451	−0.032	−0.551
ICD 2	0.228	0.778	−0.212
ICD 3	0.421	0.447	0.145
ICD 4	0.352	−0.223	0.767
ICD 5	0.446	0.347	−0.181
ICD 6	0.492	−0.148	0.094

Note: PC = Principal Component; ICD = intercusp distance. ICD 1: paracone-protocone; ICD 2: paracone-metacone; ICD 3: metacone-hypocone; ICD 4: protocone-hypocone; ICD 5: paracone-hypocone; ICD 6: protocone-metacone.

LMs were used to assess associations between molar square root area and LEH presence by zone. We set square root area as the response variable and LEH presence/absence by zone as the fixed variable. For anterior teeth, between three and eight fixed variables were tested, as some tooth zones were completely absent of LEH across all individuals.

To examine the association between LEH presence and changes to principal cusp spacing we used MLMs with a Gaussian distribution. We used PC scores as response variables and presence/absence of LEH on each anterior tooth zone as fixed variables. This statistical test allowed us to account for the repeated measures within each study individual and the hierarchical structure of our data. For each anterior tooth type, we tested between three and eight fixed variables, as some tooth zones were completely absent of LEH across all individuals.

GLMs with a binomial distribution were used to assess associations between 1) LEH presence/absence by zone and molar accessory trait expression, and 2) PC scores for principal cusp spacing and molar accessory trait expression. For the first, the fixed variables were the developmental zones of anterior teeth, and square root area was the response variable. In the second analysis, the developmental zones of anterior teeth were the fixed variables, and the presence/absence of cusp 5 or Carabelli trait were the response variables.

Based on the small effect sizes found with the variables analyzed in this study, we adopt a continuous *p*-value approach [88,89] to interpret patterns in our results that may be biologically meaningful but not traditionally associated with “statistical significance” cut offs [90]. With this approach, *p*-values greater than 0.1 are interpreted as “little to no evidence”, between 0.1 and 0.05 as “weak evidence”, between 0.05 and 0.01 as “moderate evidence”, between 0.01 and 0.001 as “strong evidence”, and less than 0.001 as “very strong evidence” [89]. We present *p*-values as well as effect size and confidence intervals to demonstrate patterns in our statistical results and interpret possible connections between variables. Similar to our previous paper [8], we use this approach due to the small sample sizes in our data, which impact the power of our analyses, and then interpret our findings within this context.

## Results

### Principal component scores

The first three Principal Components incorporated all six intercusp distances and explained 80.0% of the variation (Table 1). The first PC explained 45.9% of the variation. Each of the six cusp distances were represented as positive values with similar weights in PC1, demonstrating that the first PC is representing a coordinated change in cuspal dimensions. The four distances that explained the most variation in PC1 were the paracone-protocone (ICD1), metacone-hypocone (ICD3), protocone-metacone (ICD5), and paracone-hypocone (ICD5). The second and third PCs were explained primarily by the distance between paracone-metacone (ICD2) and protocone-hypocone (ICD4), respectively.

### LEH presence and molar crown size

**Fig 4:**
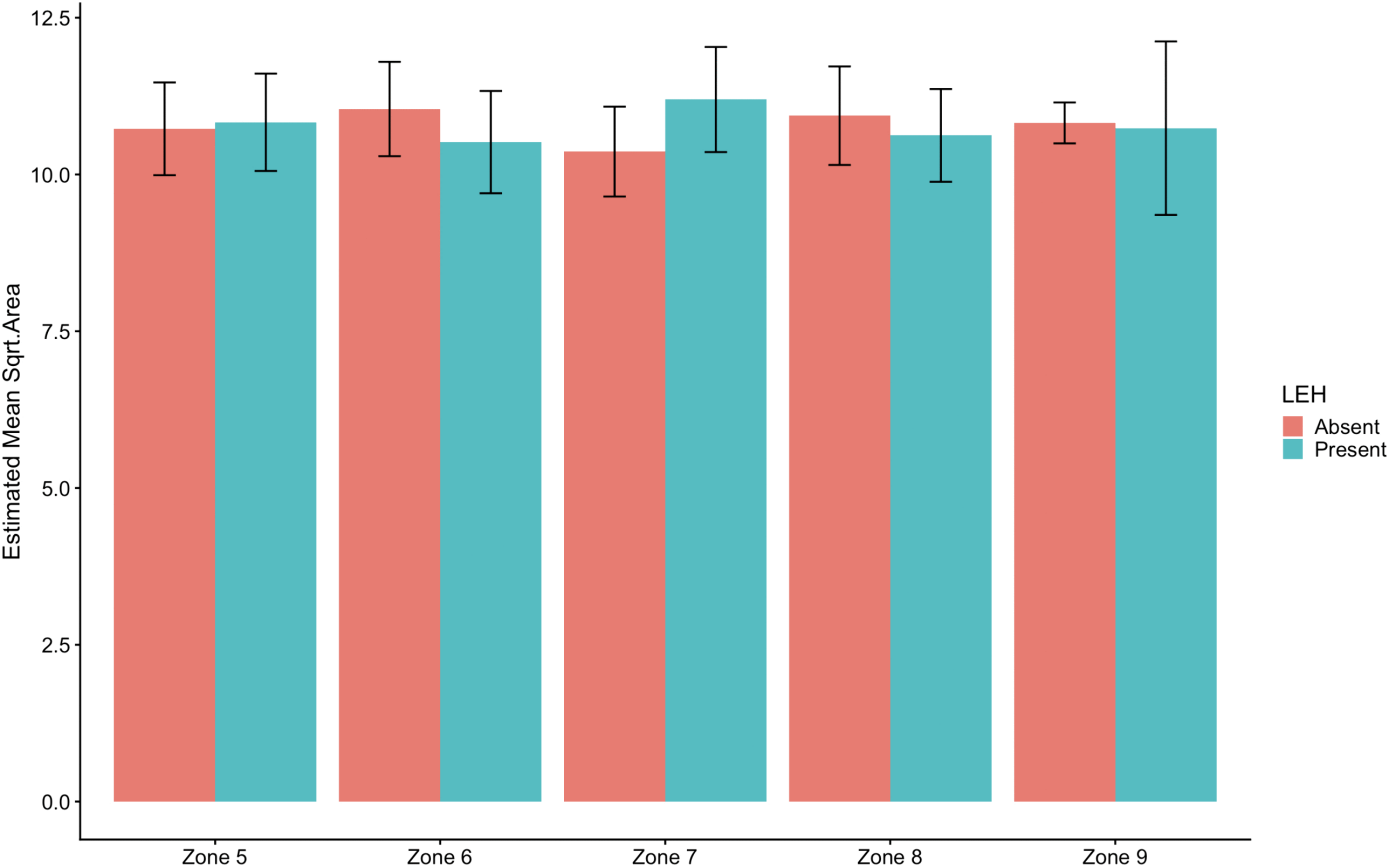
Predicted square root area when LEH is present on the upper first central incisor (A) and the upper right canine (B). In each image, the estimated marginal means are represented on the y-axis. Note: Zone 3 of the upper right canine exhibited high collinearity and was not included in statistical analyses.

One LMM showed evidence for an association between square root area and LEH presence. All other teeth showed little to no evidence for an association (S2 Table). Zone 7 of the upper left central incisor (estimated age 1.6–1.9 years) showed strong evidence (estimate = 0.830, SE = 0.303, *t* = 2.732, *p* = 0.008) for an increase in molar size when LEH was present (Fig 4).

### LEH presence and principal cusp spacing

Using MLMs, we identified evidence for several associations between PC1 scores (principal cusp spacing dimensions) and zones in the left and right central incisors, and right and left canine (S3 Table). Both the left and right central incisors show associations between LEH presence and principal cusp spacing changes. For the upper left central incisor, we found weak evidence for an association between PC2 scores and zone 8 (estimate: 0.998, SD = 0.556, *t* = 1.797, *p* = 0.078). For PC2, dimensions of ICD 2 increased with the presence of LEH in zone 8 (1.3–1.6 years). The upper right central incisor showed strong evidence of an association between PC1 scores and LEH in zone 7 (estimate: −2.236, SD = 0.734, *t* = −3.046, *p* = 0.003) and weak evidence for PC3 scores and LEH in zone 8 (estimate: 0.759, SD = 0.385, *t* = 1.970, *p* = 0.054). For PC1, dimensions of ICD 1, 3, 5, and 6 decreased with the presence of LEH in zone 7 (1.6–1.9 years). For PC3, dimensions of ICD 4 increased with the presence of LEH in zone 8 (1.3–1.6 years).

The upper left canine showed weak evidence for an association between PC1 scores and zone 5 (estimate: 1.685, SD = 0.847, *t* = 1.989, *p* = 0.052). The upper right canine showed very strong evidence for an association between PC1 scores and zone 5 (estimate: 6.193, SD = 1.669, *t* = 3.710, *p* = 0.0005). In both cases for PC1, dimensions of ICD 1, 3, 5, and 6 increased when LEH was present in zone 5 (2.1–2.5 years).

### LEH presence, cusp spacing, and molar accessory trait expression

**Fig 5:**
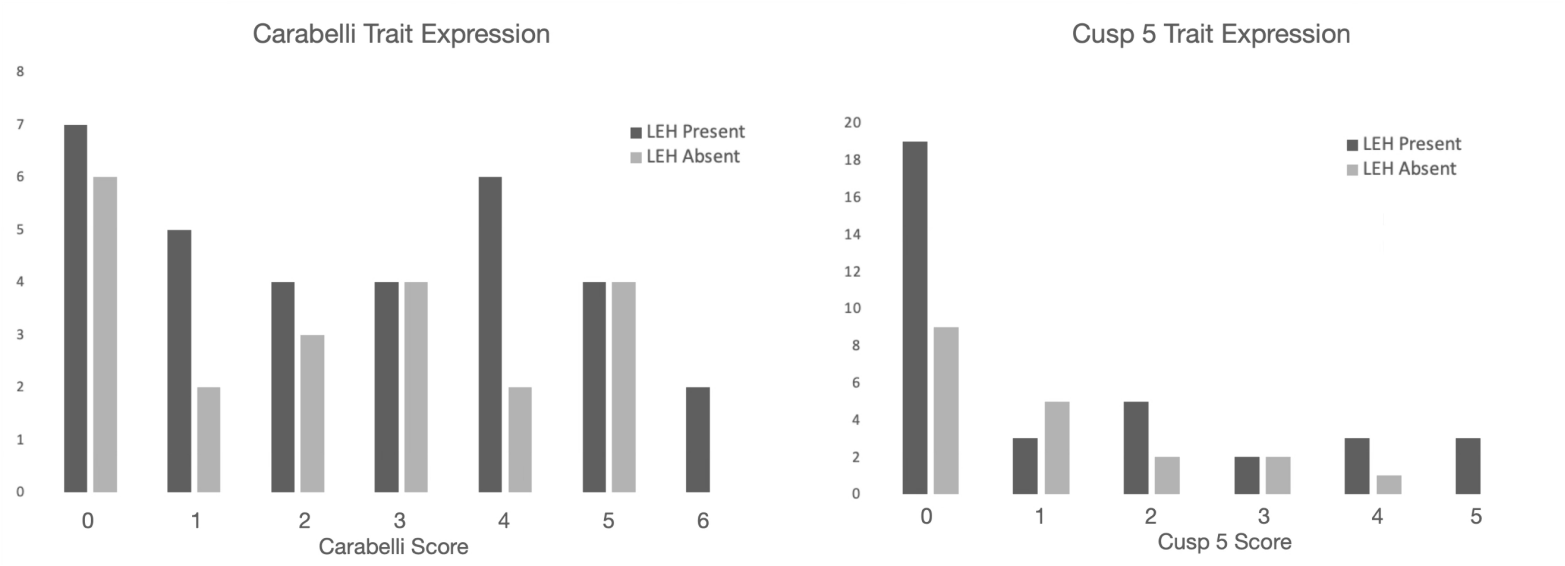
Trait expression of Carabelli trait and upper cusp 5 for individuals with LEH presence and LEH absence. For each trait, specific scores correspond to those outlined in ASUDAS [64].

We found little to no evidence for an association between LEH presence on all anterior teeth and changes to expression of Carabelli trait (S4 Table, Fig 5). Only the upper left central incisor showed weak evidence (estimate: −1.706, SD = 0.919, *t* = −1.855, *p* = 0.063) of a decrease in the presence of cusp 5 (S5 Table) associated with LEH presence in zone 5 (2.1–2.5 years).

**Fig 6:**
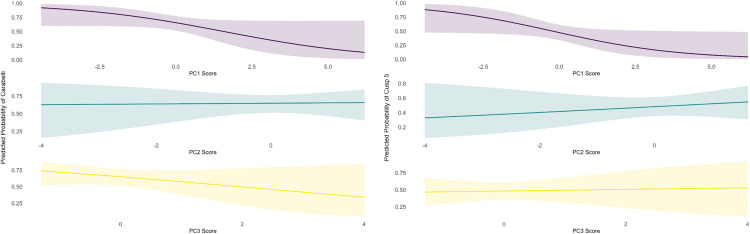
Predicted effects of Principal Components (PC1, PC2, and PC3) on the probability of expressing the Carabelli trait and upper cusp 5. Each left panel shows the marginal effect of one Principal Component score (x-axis) on the predicted probability of the Carabelli trait (y-axis), with 95% confidence intervals shaded. **Top left:** PC1 is strongly and negatively associated with the trait, with higher PC1 scores (in this case wider spacings) corresponding to a lower predicted probability of Carabelli trait presence. **Middle left:** PC2 shows little to no evidence of an effect, as the slope is near zero. **Bottom left:** PC3 shows a slight negative trend or slope, suggesting that as PC3 scores increase (in this case wider spacing), the predicted probability of Carabelli trait trends towards a decrease. Each right panel presents the marginal effect of one Principal Component score (x-axis) on the predicted probability of the cusp 5 trait (y-axis), with 95% confidence intervals shown as shaded regions. **Top right:** PC1 is strongly and negatively associated with the probability of expressing Cusp 5 meaning individuals with higher PC1 scores (in this case decreased spacings) are predicted to have a lower probability of showing the trait. **Middle right:** PC2 shows a weak positive association with Cusp 5 suggesting that as PC2 scores increase (in this case wider distances), the probability of trait expression increases. **Bottom right:** PC3 shows little to no evidence of an effect on the probability of expressing the trait, with a nearly flat slope and wide, overlapping confidence intervals.

When assessing PCs scores and trait expression probability (Fig 6), we found some evidence of associations for both cusp 5 and Carabelli trait (S6 Table). For Carabelli trait, PC1 showed weak evidence (estimate = −0.433, SE = 0.232, *z* = −1.862, *p* = 0.062) for an increase in ICD 1, 3, 5, and 6 dimensions associated with a decrease in probability of expression.

For cusp 5, PC1 shows moderate evidence (estimate = −0.484, SE = 0.242, *z* = −1.995, *p* = 0.046) for a decrease in PC1 scores and a decrease in probability of trait expression.

## Discussion

Our findings are consistent with the growing body of studies highlighting the variability of molar morphology in the presence of developmental disruptions [6–8]. At this point, the outcomes of this relationship are unpredictable, and further studies are necessary to elucidate the intricacies of when and how disruptions alter dental changes. This study represents a preliminary analysis of the association between timing of disruptions and permanent molar morphology. Our results suggest that the presence of LEH on anterior tooth zones that overlap with molar odontogenesis (between 1.6–2.5 years) shows some associations with changes to upper first molar crown size, principal cusp spacing, and accessory trait expression. Findings from this study generally follow expectations of the PCM; however, in times of disruptions, particularly early-life episodes, crown size and cusp configurations may be difficult to predict [8]. As such, our discussion explores crown size and cusp spacing independently, before interpreting their interactive effects on accessory cusps within the PCM framework.

### Crown size

We failed to find support for our prediction that LEH presence would not be associated with change in molar crown size. In the present study, we found an increase in square root area when LEH formed on the upper left central incisor between 1.6–1.9 years. This result differed from our prediction, which was based on our previous study [8], where we found no change in crown size between supplemented and non-supplemented groups. Additionally, this finding did not correspond to results from McKee and Lunz [4] who found that LEH was associated with reduced molar crown sizes in humans.

A number of studies comparing nutritional status and molar crown size have found reduced areas [56,57,91], while others concluded that molar crown size showed some degree of biological canalization where the same outcome is demonstrated regardless of environmental conditions [92,93] particularly in comparison with other dental characteristics [6]. Though our previous study on the Tezonteopan population found that mild to moderate malnutrition was not associated with a change to tooth size [8], there is precedence for molar crown size unpredictability in association with LEH. One study found that molar crown size reduced [4] while others identified both an increase [5] and decrease in molar fluctuating asymmetry associated with hypoplastic defects. This inconsistent association between LEH and changes in molar crown size may be related to the timing of developmental disruptions. Events occurring before occlusal form is set may affect cusp spacing whereas events occurring after this period may be associated with the marginal regions of the molar crown.

Although contrary to our prediction, our findings would suggest that LEH formed between 1.6–1.9 years are associated with a slight increase in first molar crown size. An increase in crown size could be caused by changes in a variety of physiological factors that regulate growth processes throughout odontogenesis. The growth (cell proliferation) and development (cell differentiation) of each tooth is assisted by growth hormone (GH) and thyroid as well as parathyroid gland activity. Garn and colleagues [17] found larger teeth in children whose mothers were experiencing hypothyroidism and diabetes. The unexpected change in tooth size may be due to alterations in GH activation, metabolic rates (thyroid gland), or vitamin D synthesis (facilitated by the parathyroid gland) necessary for adequate calcium and phosphate availability for hydroxyapatite crystal formation. Decreased GH has been found to be associated with delayed tooth formation periods which may be associated with prolonged crown and root growth [13] and possibly larger tooth dimensions. Although we do not have information about maternal endocrine health for participants included in this study, these factors may have been present and contributed to the increased molar crown size identified here. These outcomes, as well as the underlying conditions, further highlight how molar morphology may be unpredictable during disrupted periods of development.

### Cusp spacing

Our findings are consistent with the idea that this dental characteristic more varied when stressed during odontogenesis [6,8]. In our study, both central incisors and both canines with LEH formation between 1.3–2.5 years showed a relationship with changes to principal cusp spacing. We partially supported our prediction that LEH presence would be associated with a decrease in mesiodistal principal cusp dimensions. While we did find support for a change in the mesiodistal cusp dimensions, the buccolingual principal cusp dimensions – (PC1) paracone-protocone (ICD1), metacone-hypocone (ICD3), protocone-metacone (ICD5), and paracone-hypocone (ICD6) – were most often associated with the early-development of LEH. We found that the first Principal Component had similar percent contribution from all buccolingual dimensions, and all these distances showed a positive load, showing that PC1 is positively correlated with crown size. This finding suggests that, in the present sample, when LEH formed between 1.3–1.9 years of age on the upper right central incisor, dimensions of PC1 decreased together. The paracone (mesiobuccal cusp) and the protocone (mesiolingual cusp) form first. Therefore, spacing of these two cusps sets the spacing of the metacone and hypocone (distal buccal and lingual cusps respectively). Given that the PCM explains later-forming cusp spacing based on earlier-forming ones [22], the global change in dimensions found in the present study falls within expectations of this model.

Two instances showed associations between PC2 and PC3, explained by mesiodistal dimension, and LEH presence. The paracone-metacone dimension (ICD2) explained most of the variation (77.8%) in the second Principal Component whereas the protocone-hypocone dimension (ICD4) explained most of the variation (76.7%) in the third Principal Component. Although these two cusp spacings were heavily weighted, in each of these PCs there were a mix of loadings suggesting that cusp dimensions changed in slightly different ways. For example, the association between LEH presence and PC2 showed a decrease in the mesiodistal dimensions of the paracone-metacone and protocone-hypocone distances but a slight increase in the buccolingual dimensions of the metacone-hypocone and the protocone-metacone spacing. These changes in configuration follow the allometric growth patterns of molar cusps [36–38], resulting in multiple possible cusp configurations [38], some of which have been associated with PCM predictions [8,51,55].

It is understood that variation in principal cusp dimensions is the result of variation in growth processes [36–38]. In general, cusp spacing changes from initiation to mineralization as mitotic activity pushes the cusps farther apart [25]. This process appears to continue, at least to some degree, even after mineralization begins as each cusp tip on the enamel surface migrates slightly buccolingually relative to the underlying dentin horns due to the trajectory of ameloblasts [41]. These well-understood growth processes are known to be affected by homeostatic disruptions [94]. Furthermore, cusps show a prolonged developmental period [39] particularly within the grooves between cusps [95]. The length of time associated with cusp growth appears to depend on the tooth, the individual, and the population [25,39]. Cusps of the upper molars initiate and grow at different rates with the paracone and protocone completing about 40% of growth by 1.5–1.9 years [39]. While the exact timing of upper molar cusp enamel coalescence is not known, based on estimates of enamel formation timing, it seems possible that cusps are converging after this time. In the present study we found associations between LEH presence during this timeframe and changes to principal cusp dimensions. It is possible then that these early stress markers indicate disruptions that altered the tooth growth process and final tooth form in this group.

To more fully elucidate these relationships, research is needed on larger samples and in other populations. Furthermore, addition of more sensitive enamel indicators of stress, such as wider than expected perikymata spacing within a given decile, could further tease out associations between these early disruptions and changes to molar morphology.

### Accessory trait expression

In contrast to other studies that found a direct link between LEH presence and an increase in frequency and size of molar accessory trait expression [7], we found weak evidence for a decrease in cusp 5 expression associated with LEH presence. This finding, coupled with consistent dimensional loading within PC1, largely explained by buccolingual dimensions of the four principal cusps, follows expectations of the PCM [51] and provides some support for our prediction that accessory cusp expression would be associated with changes to principal cusp spacing. In some instances, with PC2 and PC3 largely explained the paracone-metacone and protocone-hypocone dimensions respectively, we found the mesiodistal dimensions decreased (PC2) or increased (PC3) to a large degree while some buccolingual dimensions increased (PC2) or decreased (PC3) slightly. As previously discussed, these changes are consistent with the allometric growth of molar cusps [36–38] For PC2, the trend showed a positive slope indicating an increase in mesiodistal dimension and an increase in probability of C5 score. For PC3, the trend showed an increase in mesiodistal spacing was accompanied by a decrease in probability of Carabelli expression. Both of these findings follow previously outlined expectations of the PCM for cusp 5 and Carabelli trait expression [8,51,55].

## Conclusions

In bioarchaeological studies, enamel hypoplasia is used to approximate the presence of perturbations that might have affected growth processes. Our study focused on the specific timing overlap between LEH formation and the periods of first maxillary permanent molar odontogenesis. Overall, our findings are in accordance with previous research showing that disruptions to the developmental environment are associated with alterations to first molar crown size, principal cusp spacing, and accessory trait expression. Results suggest there are associations between early-formed LEH on anterior dentition and molar crown size and principal cusp spacing, and to some degree accessory trait expression. The changes in cuspal dimensions associated with LEH formed after birth support the idea suggested by Butler [26] that molar cusp coalescence may occur postnatally.

Findings from the current study, and from others exploring these complex relationships [4–8] highlight the fact that genetic input is not the only factor affecting tooth morphology. These studies raise a note of caution that early developmental disruptions may impact biological outcomes to permanent dental structures in less predictable ways.

## Supporting information

S1 Table Effect size and 95% confidence interval for upper first molar crown size and principal cusp spacing. (DOCX)

S2 Table Linear mixed model for upper first molar crown size and liner enamel hypoplasia presence by tooth zone. (DOCX)

S3 Table Linear mixed model for upper first molar Principal Component Analysis of principal cusp spacing and liner enamel hypoplasia presence by tooth zone. (DOCX)

S4 Table Generalized linear model for Carabelli trait and LEH presence by tooth zones. (DOCX)

S5 Table Generalized linear model for Cusp 5 and LEH presence by tooth zones. (DOCX)

S6 Table Generalized linear model for probability of Carabelli trait and cusp 5 expression based on estimated Principal Component scores. (DOCX)
