# Supplementary material for: Tracing early life stress in human molar morphology: Associations between linear enamel hypoplasia and maxillary first molar form
Source: PLoS One. 2026 Jul 29;21(7):e0354698. doi: 10.1371/journal.pone.0354698 (PMC13419181; doi:10.1371/journal.pone.0354698)
Supplement: S1 Table — (DOCX) [file pone.0354698.s001.docx]

**S1 Table. Effect size and 95% confidence interval for upper first molar crown size and principal cusp spacing.**

| **Variable** | **Effect size*** | **95% Confidence Interval** |
| --- | --- | --- |
| M1 crown size | 0.00879 (very small) | -0.289; 0.292 |
| ICD 1 | 0.0860 (very small) | -0.015; 0.029 |
| ICD 2 | 0.0606 (very small) | -0.017; 0.031 |
| ICD 3 | 0.186 (small) | -0.002; 0.041 |
| ICD 4 | 0.181 (small) | -0.002; 0.032 |
| ICD 5 | 0.176 (small) | -0.003; 0.040 |
| ICD 6 | 0.246 (small) | -0.003; 0.042 |

*Effect size and magnitude based on Cohen [1] where |r| < 0.01: very small effect; |r|=0.3: medium effect, and |r|=0.05: large effect

Reference list for supporting information:

1. Cohen J. Statistical Power analysis for the behavioral sciences. (2nd edition). New Jersey: Erlbaum; 1988
