# Supplementary material for: Tracing early life stress in human molar morphology: Associations between linear enamel hypoplasia and maxillary first molar form
Source: PLoS One. 2026 Jul 29;21(7):e0354698. doi: 10.1371/journal.pone.0354698 (PMC13419181; doi:10.1371/journal.pone.0354698)
Supplement: S2 Table — (DOCX) [file pone.0354698.s002.docx]

**S2 Table. Linear mixed model for upper first molar crown size and liner enamel hypoplasia presence by tooth zone.**

| **Tooth** | **Zone** | **Estimate** | **SE** | **t-value** | **Pr(>ItI)** |
| --- | --- | --- | --- | --- | --- |
| ULC | Intercept | 10.812 | 0.096 | 111.609 | <0.001 |
|  | Zone 5 | -0.466 | 0.352 | -1.322 | 0.192 |
|  | Zone 6 | - | - | - | - |
| URC | Intercept | 10.810 | 0.085 | 126.479 | <0.001 |
|  | Zone 5 | 0.191 | 0.356 | 0.539 | 0.592 |
|  | Zone 6 | - | - | - | - |
| ULI2 | Intercept | 10.746 | 0.098 | 109.589 | <0.001 |
|  | Zone 5 | 0.238 | 0.462 | 0.515 | 0.609 |
|  | Zone 6 | 0.178 | 0.365 | 0.488 | 0.628 |
| URI2 | Intercept | 10.777 | 0.093 | 114.886 | <0.001 |
|  | Zone 5 | -0.072 | 0.689 | -0.106 | 0.916 |
|  | Zone 6 | - | - | - | - |
| URI1 | Intercept | 10.730 | 0.109 | 98.431 | <0.001 |
|  | Zone 5 | -0.120 | 0.302 | -0.398 | 0.693 |
|  | Zone 6 | 0.329 | 0.323 | 1.019 | 0.313 |
|  | Zone 7 | 0.376 | 0.370 | 1.017 | 0.314 |
|  | Zone 8 | -0.267 | 0.359 | -0.744 | 0.461 |
|  | Zone 9 | 0.281 | 0.481 | 0.584 | 0.562 |
| ULI1 | Intercept | 10.775 | 0.110 | 97.877 | <0.001 |
|  | Zone 5 | 0.104 | 0.243 | 0.428 | 0.670 |
|  | Zone 6 | -0.528 | 0.315 | -1.673 | 0.100 |
|  | Zone 7 | 0.830 | 0.303 | 2.732 | 0.008*** |
|  | Zone 8 | -0.314 | 0.258 | -1.218 | 0.229 |
|  | Zone 9 | -0.083 | 0.699 | -0.102 | 0.905 |

Note: Blank cells indicate that no individuals exhibited LEH in that zone; therefore, variance could not be estimated and the linear mixed‐effects model could not be fitted for those zones. Bold indicates very strong evidence; *** indicates strong evidence; ** indicates moderate evidence; * indicates weak evidence
