# Supplementary material for: Tracing early life stress in human molar morphology: Associations between linear enamel hypoplasia and maxillary first molar form
Source: PLoS One. 2026 Jul 29;21(7):e0354698. doi: 10.1371/journal.pone.0354698 (PMC13419181; doi:10.1371/journal.pone.0354698)
Supplement: S3 Table — (DOCX) [file pone.0354698.s003.docx]

**S3 Table. Linear mixed model for upper first molar Principal Component Analysis of principal cusp spacing and liner enamel hypoplasia presence by tooth zone.**

| **Tooth** | **PC** | **Zone** | **Estimate** | **SE** | **t-value** | **Pr(>ItI)** |
| --- | --- | --- | --- | --- | --- | --- |
| ULC | PC1 | Intercept | -0.129 | 0.232 | -0.556 | 0.581 |
|  |  | Zone 5 | 1.685 | 0.847 | 1.989 | 0.052* |
|  |  | Zone 6 | 0.245 | 1.645 | 0.149 | 0.882 |
|  | PC2 | Intercept | 0.016 | 0.151 | 0.109 | 0.914 |
|  |  | Zone 5 | 0.964 | 1.068 | 0.903 | 0.371 |
|  |  | Zone 6 | -0.464 | 0.550 | -0.843 | 0.403 |
|  | PC3 | Intercept | 0.022 | 0.140 | 0.160 | 0.873 |
|  |  | Zone 5 | -1.019 | 0.992 | -1.027 | 0.309 |
|  |  | Zone 6 | -0.048 | 0.511 | -0.096 | 0.924 |
| URC | PC1 | Intercept | -0.124 | 0.204 | -0.609 | 0.545 |
|  |  | Zone 5 | 6.193 | 1.669 | 3.710 | **0.0005** |
|  |  | Zone 6 | 0.131 | 0.859 | 0.153 | 0.878 |
|  | PC2 | Intercept | 0.035 | 0.149 | 0.239 | 0.812 |
|  |  | Zone 5 | -0.973 | 1.224 | -0.795 | 0.430 |
|  |  | Zone 6 | -0.239 | 0.631 | -0.381 | 0.705 |
|  | PC3 | Intercept | 0.002 | 0.138 | 0.015 | 0.988 |
|  |  | Zone 5 | 1.467 | 1.127 | 1.301 | 0.199 |
|  |  | Zone 6 | -0.395 | 0.580 | -0.681 | 0.499 |
| ULI2 | PC1 | Intercept | -0.012 | 0.242 | -0.505 | 0.961 |
|  |  | Zone 5 | 0.134 | 1.145 | 0.188 | 0.907 |
|  |  | Zone 6 | 0.049 | 0.904 | 0.054 | 0.957 |
|  | PC2 | Intercept | 0.018 | 0.151 | 0.121 | 0.904 |
|  |  | Zone 5 | -0.911 | 0.716 | -1.271 | 0.209 |
|  |  | Zone 6 | 0.348 | 0.566 | 0.615 | 0.541 |
|  | PC3 | Intercept | -0.001 | 0.141 | -0.012 | 0.990 |
|  |  | Zone 5 | 0.434 | 0.669 | 0.648 | 0.520 |
|  |  | Zone 6 | -0.241 | 0.529 | -0.457 | 0.650 |
| URI2 | PC1 | Intercept | -0.019 | 0.229 | -0.084 | 0.933 |
|  |  | Zone 5 | 1.044 | 1.684 | 0.620 | 0.538 |
|  |  | Zone 6 | - | - | - | - |
|  | PC2 | Intercept | 0.012 | 0.145 | 0.086 | 0.932 |
|  |  | Zone 5 | -0.678 | 1.070 | -0.634 | 0.529 |
|  |  | Zone 6 | - | - | - | - |
|  | PC3 | Intercept | -0.000 | 0.000 | 0.000 | 1.000 |
|  |  | Zone 5 | 0.001 | 0.001 | 0.001 | 0.999 |
|  |  | Zone 6 | - | - | - | - |
| URI1 | PC1 | Intercept | -0.032 | 0.266 | -0.123 | 0.902 |
|  |  | Zone 5 | 0.712 | 0.589 | 1.209 | 0.232 |
|  |  | Zone 6 | 0.755 | 0.762 | 0.991 | 0.326 |
|  |  | Zone 7 | -2.236 | 0.734 | -3.046 | 0.003*** |
|  |  | Zone 8 | 0.230 | 0.623 | 0.370 | 0.712 |
|  |  | Zone 9 | 0.034 | 1.691 | 0.021 | 0.983 |
|  | PC2 | Intercept | 0.045 | 0.183 | 0.251 | 0.803 |
|  |  | Zone 5 | -0.100 | 0.405 | -0.247 | 0.806 |
|  |  | Zone 6 | -0.196 | 0.525 | -0.374 | 0.710 |
|  |  | Zone 7 | 0.093 | 0.505 | 0.186 | 0.854 |
|  |  | Zone 8 | -0.179 | 0.429 | -0.415 | 0.677 |
|  |  | Zone 9 | 1.356 | 1.164 | 1.165 | 0.250 |
|  | PC3 | Intercept | -0.088 | 0.164 | -0.540 | 0.591 |
|  |  | Zone 5 | -0.009 | 0.364 | -0.273 | 0.785 |
|  |  | Zone 6 | -0.128 | 0.471 | -0.274 | 0.785 |
|  |  | Zone 7 | -0.044 | 0.453 | 0.099 | 0.921 |
|  |  | Zone 8 | 0.759 | 0.385 | 1.970 | 0.054* |
|  |  | Zone 9 | -0.024 | 1.045 | -0.024 | 0.981 |
| ULI1 | PC1 | Intercept | 0.116 | 0.259 | 0.449 | 0.655 |
|  |  | Zone 5 | 1.203 | 0.719 | 1.672 | 0.101 |
|  |  | Zone 6 | -1.132 | 0.770 | -1.470 | 0.148 |
|  |  | Zone 7 | -0.975 | 0.882 | -1.106 | 0.274 |
|  |  | Zone 8 | -0.047 | 0.856 | -0.056 | 0.956 |
|  |  | Zone 9 | -0.945 | 1.146 | -0.824 | 0.414 |
|  | PC2 | Intercept | -0.013 | 0.168 | -0.079 | 0.937 |
|  |  | Zone 5 | 0.042 | 0.467 | 0.091 | 0.927 |
|  |  | Zone 6 | -0.103 | 0.500 | -0.207 | 0.836 |
|  |  | Zone 7 | -0.641 | 0.572 | -1.121 | 0.267 |
|  |  | Zone 8 | 0.998 | 0.556 | 1.797 | 0.078* |
|  |  | Zone 9 | -0.550 | 0.744 | -0.754 | 0.454 |
|  | PC3 | Intercept | -0.032 | 0.159 | -0.202 | 0.840 |
|  |  | Zone 5 | 0.330 | 0.443 | 0.746 | 0.459 |
|  |  | Zone 6 | -0.304 | 0.474 | -0.642 | 0.524 |
|  |  | Zone 7 | -0.124 | 0.543 | -0.229 | 0.802 |
|  |  | Zone 8 | 0.320 | 0.527 | 0.607 | 0.547 |
|  |  | Zone 9 | -0.021 | 0.706 | -0.031 | 0.976 |

Note: Blank cells indicate that no individuals exhibited LEH in that zone; therefore, variance could not be estimated and the linear mixed‐effects model could not be fitted for those zones. Bold indicates very strong evidence; *** indicates strong evidence; ** indicates moderate evidence; * indicates weak evidence
