# Supplementary material for: Tracing early life stress in human molar morphology: Associations between linear enamel hypoplasia and maxillary first molar form
Source: PLoS One. 2026 Jul 29;21(7):e0354698. doi: 10.1371/journal.pone.0354698 (PMC13419181; doi:10.1371/journal.pone.0354698)
Supplement: S4 Table — (DOCX) [file pone.0354698.s004.docx]

**S4 Table. Generalized linear model for Carabelli trait and LEH presence by tooth zones.**

| **Tooth** |  | **Estimate** | **SE** | **z value** | **Pr(>IzI)** |
| --- | --- | --- | --- | --- | --- |
| ULC | Intercept | 0.818 | 0.310 | 2.640 | 0.008 |
|  | Zone 5 | -18.384 | 1978.090 | -0.009 | 0.992 |
|  | Zone 6 | - | - | - | - |
| URC | Intercept | 0.581 | 0.286 | 2.032 | 0.042 |
|  | Zone 5 | <0.001 | <0.001 | -0.008 | 0.993 |
|  | Zone 6 | - | - | - | - |
| ULI2 | Intercept | 0.510 | 0.298 | 1.713 | 0.086 |
|  | Zone 5 | 15.935 | 1383.993 | 0.012 | 0.990 |
|  | Zone 6 | 0.182 | 1.260 | 0.145 | 0.885 |
| URI2 | Intercept | 0.581 | 0.286 | 2.032 | 0.042 |
|  | Zone 5 | 14.984 | 1455.397 | 0.010 | 0.991 |
|  | Zone 6 | - | - | - | - |
| ULI1 | Intercept | 0.587 | 0.357 | 1.644 | 1.000 |
|  | Zone 5 | -0.051 | 0.804 | -0.064 | 0.949 |
|  | Zone 6 | 0.294 | 1.086 | 0.027 | 0.978 |
|  | Zone 7 | 1.324 | 1.241 | 1.067 | 0.286 |
|  | Zone 8 | -0.341 | 0.836 | -0.408 | 0.683 |
|  | Zone 9 | -15.812 | 1455.397 | -0.011 | 0.991 |
| URI1 | Intercept | 0.452 | 0.341 | 1.322 | 0.186 |
|  | Zone 5 | -1.550 | 1.204 | -1.288 | 0.198 |
|  | Zone 6 | 36.259 | 4115.408 | 0.009 | 0.993 |
|  | Zone 7 | -0.452 | 1.455 | -0.311 | 0.756 |
|  | Zone 8 | 17.640 | 2723.555 | 0.006 | 0.995 |
|  | Zone 9 | -18.092 | 2723.555 | -0.007 | 0.995 |

Note: Blank cells indicate that no individuals exhibited LEH in that zone; therefore, variance could not be estimated and the linear mixed‐effects model could not be fitted for those zones. Bold indicates very strong evidence; *** indicates strong evidence; ** indicates moderate evidence; * indicates weak evidence
