# Supplementary material for: Tracing early life stress in human molar morphology: Associations between linear enamel hypoplasia and maxillary first molar form
Source: PLoS One. 2026 Jul 29;21(7):e0354698. doi: 10.1371/journal.pone.0354698 (PMC13419181; doi:10.1371/journal.pone.0354698)
Supplement: S5 Table — (DOCX) [file pone.0354698.s005.docx]

**S5 Table. Generalized linear model for Cusp 5 and LEH presence by tooth zones.**

| **Tooth** |  | **Estimate** | **SE** | **z value** | **Pr(>IzI)** |
| --- | --- | --- | --- | --- | --- |
| ULC | Intercept | -0.040 | 0.285 | -0.143 | 0.886 |
|  | Zone 5 | 15.606 | 1455.397 | 0.011 | 0.991 |
|  | Zone 6 | - | - | - | - |
| URC | Intercept | <0.001 | <0.001 | 0.000 | 1.000 |
|  | Zone 5 | <0.001 | <0.001 | -0.010 | 0.992 |
|  | Zone 6 | - | - | - | - |
| ULI2 | Intercept | <0.001 | <0.001 | 0.000 | 1.000 |
|  | Zone 5 | <0.001 | <0.001 | 0.009 | 0.993 |
|  | Zone 6 | <0.001 | <0.001 | -0.009 | 0.993 |
| URI2 | Intercept | -0.037 | 0.274 | -0.137 | 0.891 |
|  | Zone 5 | -15.528 | 1455.397 | -0.011 | 0.991 |
|  | Zone 6 | - | - | - | - |
| ULI1 | Intercept | 0.169 | 0.341 | 0.050 | 0.960 |
|  | Zone 5 | -1.706 | 0.919 | -1.855 | 0.063* |
|  | Zone 6 | 0.928 | 1.091 | 0.848 | 0.396 |
|  | Zone 7 | 0.483 | 0.981 | 0.493 | 0.622 |
|  | Zone 8 | 0.923 | 0.868 | 1.063 | 0.287 |
|  | Zone 9 | -16.506 | 1455.397 | -0.011 | 0.991 |
| URI1 | Intercept | -0.095 | 0.318 | -0.300 | 0.764 |
|  | Zone 5 | -1.123 | 0.994 | -1.131 | 0.258 |
|  | Zone 6 | 0.659 | 0.997 | 0.661 | 0.508 |
|  | Zone 7 | 0.934 | 1.144 | 0.816 | 0.414 |
|  | Zone 8 | -0.583 | 1.094 | -0.533 | 0.594 |
|  | Zone 9 | 1.333 | 1.481 | 0.900 | 0.368 |

Note: Blank cells indicate that no individuals exhibited LEH in that zone; therefore, variance could not be estimated and the linear mixed‐effects model could not be fitted for those zones. Bold indicates very strong evidence; *** indicates strong evidence; ** indicates moderate evidence; * indicates weak evidence
