# Supplementary material for: Tracing early life stress in human molar morphology: Associations between linear enamel hypoplasia and maxillary first molar form
Source: PLoS One. 2026 Jul 29;21(7):e0354698. doi: 10.1371/journal.pone.0354698 (PMC13419181; doi:10.1371/journal.pone.0354698)
Supplement: S6 Table — (DOCX) [file pone.0354698.s006.docx]

**S6 Table. Generalized linear model for probability of Carabelli trait and cusp 5 expression based on estimated Principal Component scores.**

| **Trait** | **PC** | **Estimate** | **SE** | **z value** | **Pr(>IzI)** |
| --- | --- | --- | --- | --- | --- |
| Carabelli | PC1 | -0.433 | 0.232 | -1.862 | 0.062* |
|  | PC2 | 0.035 | 0.279 | 0.127 | 0.898 |
|  | PC3 | -0.354 | 0.315 | -1.125 | 0.260 |
| Cusp 5 | PC1 | -0.484 | 0.242 | -1.995 | 0.046** |
|  | PC2 | 0.177 | 0.277 | 0.640 | 0.522 |
|  | PC3 | 0.031 | 0.293 | 0.108 | 0.914 |

Note: Bold indicates very strong evidence; *** indicates strong evidence; ** indicates moderate evidence; * indicates weak evidence
