# Supplementary material for: Tracing early life stress in human molar morphology: Associations between linear enamel hypoplasia and maxillary first molar form
Source: PLoS One. 2026 Jul 29;21(7):e0354698. doi: 10.1371/journal.pone.0354698 (PMC13419181; doi:10.1371/journal.pone.0354698)
Supplement: Attachment — Submitted filename: Response to Reviewers.docx [file pone.0354698.s008.docx]

Ajaya Bhattarai, Ph.D.

Academic Editor

PLOS One

Dear Dr. Bhattarai and PLOS One Manuscript Editors and Reviewers,

My colleagues and I are pleased to submit our revised manuscript titled *Tracing early life stress in human molar morphology: Associations between linear enamel hypoplasia and maxillary first molar form*. We thank you, the Academic Editor, and our reviewers for your thoughtful and insightful comments.

Most of the comments we received related to clarifying aspects of the methods and results, and editing citations. These comments were highly valuable to help clarify important points within this manuscript. Below please find our responses to the comments made by the Academic Editor and each reviewer.

**Academic Editor:**

Comment 1: Please ensure that your manuscript meets PLOS ONE's style requirements, including those for file naming.

Response: The manuscript and file names are in accordance with PLOS ONE’s style requirements.

Comment 2: In your manuscript, please provide additional information regarding the specimens used in your study. Ensure that you have reported human remain specimen numbers and complete repository information, including museum name and geographic location.

Response: A supplemental table (new S1 Table) was added with participation identification numbers as well as the institution and location where the casts are housed. However, after resubmission, we received an email saying that all human subject information should be de-identified. Because there is no other subject identification, this table was removed from the supporting information. We changed lines 316-317 to state where the casts were housed.

Comment 3: Please state what role the funders took in the study.

Response: The following amended Role of Funder statement has been added to the cover letter: The funders had no role in study design, data collection and analysis, decision to publish, or preparation of the manuscript.

Comment 4: In the online submission form you indicate that your data is not available for proprietary reasons and have provided a contact point for accessing this data. Please note that your current contact point is a co-author on this manuscript. According to our Data Policy, the contact point must not be an author on the manuscript and must be an institutional contact, ideally not an individual. Please revise your data statement to a non-author institutional point of contact, such as a data access or ethics committee, and send this to us via return email. Please also include contact information for the third party organization, and please include the full citation of where the data can be found.

Response: These data have been added to OSF with the following link: https://osf.io/zh9k8/overview?view_only=6a646d054c54462cbac5c3f5559929b7

Comment 5: Please amend the manuscript submission data (via Edit Submission) to include author Debbie Guatelli-Steinberg.

Response: Debbie Guatelli-Steinberg has been amended via Edit Submission.

Comment 6: Please amend your authorship list in your manuscript file to include author Debra Guatelli-Steinberg.

Response: Debbie Guatelli-Steinberg has been amended in the manuscript author list.

Comment 7: Please include your full ethics statement in the ‘Methods’ section of your manuscript file. In your statement, please include the full name of the IRB or ethics committee who approved or waived your study, as well as whether or not you obtained informed written or verbal consent. If consent was waived for your study, please include this information in your statement as well.

Response: Lines 305-307 have been updated as follows: This portion of the study was approved by the Mexican National Institute of Nutrition and Hampshire College’s Institutional Review Boards (IRB) [63]. Informed written consent was obtained prior to participation in the study.

Comment 8: If the reviewer comments include a recommendation to cite specific previously published works, please review and evaluate these publications to determine whether they are relevant and should be cited. There is no requirement to cite these works unless the editor has indicated otherwise.

Response: Thank you for this comment. The suggestion from Reviewer 2 was helpful in clarifying that the statement was made based on a different study. Therefore, lines 55-56 have been changed to accurately reflect what the cited article presented.

Comment 9: Please review your reference list to ensure that it is complete and correct. If you have cited papers that have been retracted, please include the rationale for doing so in the manuscript text, or remove these references and replace them with relevant current references. Any changes to the reference list should be mentioned in the rebuttal letter that accompanies your revised manuscript. If you need to cite a retracted article, indicate the article’s retracted status in the References list and also include a citation and full reference for the retraction notice.

Response: All citations have been updated to reflect expectations of the journal.

**Reviewer 1:**

Comment 1: the discussion of cusp tip spacing throughout the manuscript could use some minor clarification. I think I understand what the paper is saying but consistently differentiating and referencing whether the cusp tips of the EDJ or the enamel surface would help clarify some of the confusion I had reading through this section. A figure would help greatly.

Response: Thank you for your comment. Lines 173-174, 221, and 608 have been changed to clarify this point. Fig 3 is meant to demonstrate the locations of each intercusp distance measurement. To help clarify this, “of enamel cusp tips” has been added to line 387 to communicate that we measured cusp spacing using enamel cusp tips rather than from the EDJ.

Comment 2: measuring inter-cusp distances on physical casts is not unheard of but is typically done with a labor intensive digital approach. Does observer error data suggest these date are accurate? I assume the tips were marked prior to measuring (did I miss this). If so, a statement should be added to this effect.

Response: Thank you for allowing us to clarify. Lines 322-324 were added with two citations showing there are no statistically significant differences between cast and image measurements. Because this added two citations, numbering was updated throughout the rest of the manuscript and in the references cited section. Line 382 was altered to show that cusp tips were marked prior to measurement.

Comment 3: Although perhaps not needed for this study, I do wonder how visible the LEH defects (not discolorations) are on the casts. There are numerous casted populations like the one used here. Some recent studies have used LEH data collected form casts I have seen clear LEH on casts myself. I wonder how much sensitivity is lost when collecting such data. This would be a nice addition, or perhaps for a tech note elsewhere.

Response: Thank you for your comment and allowing us to clarify within the manuscript. LEH were recorded from the actual dentition, not the casts, by AHG. Lines 328-329 have been changed to clarify the source of these data.

Comment 4: Using a breakpoint of 1 for Carabelli's trait is stated as necessary due to the low sample frequency of this trait. Is it possible to run the analyses using true cusp forms of this trait only? What are the ordinal scores for Carabelli's and cusp 5 in this sample? That would be useful information to include somewhere.

Response: Thank you for this suggestion. A figure (new fig 5) has been added, showing the ordinal breakdown of trait expression for individuals with and without LEH for each Carabelli and C5 traits.

**Reviewer 2:**

Comment 1: I was struck by the use of the term “adult teeth,” especially since the study focuses on a specific stage of their developmental process. I would perhaps retain the more commonly used and universally recognizable terminology, namely “permanent teeth.”

Response: Thank you for your comment. Lines 29, 34, 51 and 60 have been changed from “adult” to “permanent”.

Comment 2: The citation of the work by Skinner and colleagues should be slightly reformulated. It is not Skinner et al. themselves who identify “a noticeable decrease in heritability of tooth size over the last few decades”; rather, in a study based on pigs, they discuss and report observations made by other authors. It may be sufficient to rephrase the sentence by stating that Skinner and colleagues, referring to previous studies, comment on this decrease.

Response: Thank you very much for pointing this out. Lines 55-56 have been changed to reflect the fact that Skinner and colleagues are basing this statement based on patterns observed from previous studies.

Comment 3: In any case, the Skinner reference in the bibliography contains a typo error in the parentheses, and the scientific name of Sus scrofa should follow standard nomenclature: it should be in italic, with “scrofa” in lower case.

Response: Thank you for your comment. Lines 687-689 have been changed.

Comment 4: Finally, the bibliography should be reformatted as the final version of the article will be ready.

Response: All citations have been reformatted to match expectations of the journal.

Thank you for consideration of our revised manuscript and for the opportunity to revise our submitted version.

Sincerely,

Erin C. Blankenship-Sefczek, Ph.D., Alan H. Goodman Ph.D., Mark Hubbe Ph.D., John P. Hunter Ph.D., Timothy M. Sefczek Ph.D., and Debbie Guatelli-Steinberg Ph.D.
