# Supplementary material for: Tracing early life stress in human molar morphology: Associations between linear enamel hypoplasia and maxillary first molar form
Source: PLoS One. 2026 Jul 29;21(7):e0354698. doi: 10.1371/journal.pone.0354698 (PMC13419181; doi:10.1371/journal.pone.0354698)
Supplement: Peer review history

# Peer review: Decision Letter 0

**DOI:** 10.1371/journal.pone.0354698.r001

**Authors:** Ajaya Bhattarai

15 Jun 2026

PONE-D-26-21105

Tracing early life stress in human molar morphology: Associations between linear enamel hypoplasia and maxillary first molar form

PLOS One

Dear Dr. Blankenship-Sefczek,

Thank you for submitting your manuscript to PLOS ONE. After careful consideration, we feel that it has merit but does not fully meet PLOS ONE’s publication criteria as it currently stands. Therefore, we invite you to submit a revised version of the manuscript that addresses the points raised during the review process.

Please submit your revised manuscript by Jul 15 2026 11:59PM. If you will need more time than this to complete your revisions, please reply to this message or contact the journal office at plosone@plos.org. When you're ready to submit your revision, log on to https://www.editorialmanager.com/pone/ and select the 'Submissions Needing Revision' folder to locate your manuscript file.

Please include the following items when submitting your revised manuscript:

- A letter that responds to each point raised by the academic editor and reviewer(s). You should upload this letter as a separate file labeled 'Response to Reviewers'.
- A marked-up copy of your manuscript that highlights changes made to the original version. You should upload this as a separate file labeled 'Revised Manuscript with Track Changes'.
- An unmarked version of your revised paper without tracked changes. You should upload this as a separate file labeled 'Manuscript'.

If you would like to make changes to your financial disclosure, please include your updated statement in your cover letter. Guidelines for resubmitting your figure files are available below the reviewer comments at the end of this letter.

If applicable, we recommend that you deposit your laboratory protocols in protocols.io to enhance the reproducibility of your results. Protocols.io assigns your protocol its own identifier (DOI) so that it can be cited independently in the future. For instructions see: https://journals.plos.org/plosone/s/submission-guidelines#loc-laboratory-protocols. Additionally, PLOS ONE offers an option for publishing peer-reviewed Lab Protocol articles, which describe protocols hosted on protocols.io. Read more information on sharing protocols at https://plos.org/protocols?utm_medium=editorial-email&utm_source=authorletters&utm_campaign=protocols.

As the corresponding author, your ORCID iD is verified in the submission system and will appear in the published article. PLOS supports the use of ORCID, and we encourage all coauthors to register for an ORCID iD and use it as well. Please encourage your coauthors to verify their ORCID iD within the submission system before final acceptance, as unverified ORCID iDs will not appear in the published article. *Only*  the individual author can complete the verification step; PLOS staff *cannot*  verify ORCID iDs on behalf of authors.

We look forward to receiving your revised manuscript.

Kind regards,

Ajaya Bhattarai

Academic Editor

PLOS One

Journal Requirements:

When submitting your revision, we need you to address these additional requirements.

1. Please ensure that your manuscript meets PLOS ONE's style requirements, including those for file naming. The PLOS ONE style templates can be found at

https://journals.plos.org/plosone/s/file?id=wjVg/PLOSOne_formatting_sample_main_body.pdf and

https://journals.plos.org/plosone/s/file?id=ba62/PLOSOne_formatting_sample_title_authors_affiliations.pdf

2. In your manuscript, please provide additional information regarding the specimens used in your study. Ensure that you have reported human remain specimen numbers and complete repository information, including museum name and geographic location.

If permits were required, please ensure that you have provided details for all permits that were obtained, including the full name of the issuing authority, and add the following statement:

'All necessary permits were obtained for the described study, which complied with all relevant regulations.'

If no permits were required, please include the following statement:

'No permits were required for the described study, which complied with all relevant regulations.'

For more information on PLOS One's requirements for paleontology and archeology research, see https://journals.plos.org/plosone/s/submission-guidelines#loc-paleontology-and-archaeology-research.

3. Thank you for stating the following financial disclosure:

“This work was supported in part by grant RO3 DE08607 from the National Institutes of Health and by the 3M Corporation, St. Paul.”

Please state what role the funders took in the study.  If the funders had no role, please state: "The funders had no role in study design, data collection and analysis, decision to publish, or preparation of the manuscript."

If this statement is not correct you must amend it as needed.

Please include this amended Role of Funder statement in your cover letter; we will change the online submission form on your behalf.

4. In the online submission form you indicate that your data is not available for proprietary reasons and have provided a contact point for accessing this data. Please note that your current contact point is a co-author on this manuscript. According to our Data Policy, the contact point must not be an author on the manuscript and must be an institutional contact, ideally not an individual. Please revise your data statement to a non-author institutional point of contact, such as a data access or ethics committee, and send this to us via return email. Please also include contact information for the third party organization, and please include the full citation of where the data can be found.

5. Please amend the manuscript submission data (via Edit Submission) to include author Debbie Guatelli-Steinberg.

6. Please amend your authorship list in your manuscript file to include author Debra Guatelli-Steinberg.

7. Please include your full ethics statement in the ‘Methods’ section of your manuscript file. In your statement, please include the full name of the IRB or ethics committee who approved or waived your study, as well as whether or not you obtained informed written or verbal consent. If consent was waived for your study, please include this information in your statement as well.

8. If the reviewer comments include a recommendation to cite specific previously published works, please review and evaluate these publications to determine whether they are relevant and should be cited. There is no requirement to cite these works unless the editor has indicated otherwise.

9. Please review your reference list to ensure that it is complete and correct. If you have cited papers that have been retracted, please include the rationale for doing so in the manuscript text, or remove these references and replace them with relevant current references. Any changes to the reference list should be mentioned in the rebuttal letter that accompanies your revised manuscript. If you need to cite a retracted article, indicate the article’s retracted status in the References list and also include a citation and full reference for the retraction notice.

Additional Editor Comments:

The minor revision is required.

[Note: HTML markup is below. Please do not edit.]

Reviewers' comments:

Reviewer's Responses to Questions

**Comments to the Author**

1. Is the manuscript technically sound, and do the data support the conclusions?

The manuscript must describe a technically sound piece of scientific research with data that supports the conclusions. Experiments must have been conducted rigorously, with appropriate controls, replication, and sample sizes. The conclusions must be drawn appropriately based on the data presented.

Reviewer #1: Yes

Reviewer #2: Yes

**********

2. Has the statistical analysis been performed appropriately and rigorously?

Reviewer #1: Yes

Reviewer #2: Yes

**********

3. Have the authors made all data underlying the findings in their manuscript fully available?

The PLOS Data policy requires authors to make all data underlying the findings described in their manuscript fully available without restriction, with rare exception (please refer to the Data Availability Statement in the manuscript PDF file). The data should be provided as part of the manuscript or its supporting information, or deposited to a public repository. For example, in addition to summary statistics, the data points behind means, medians and variance measures should be available. If there are restrictions on publicly sharing data—e.g. participant privacy or use of data from a third party—those must be specified.

Reviewer #1: Yes

Reviewer #2: Yes

**********

4. Is the manuscript presented in an intelligible fashion and written in standard English?

PLOS ONE does not copyedit accepted manuscripts, so the language in submitted articles must be clear, correct, and unambiguous. Any typographical or grammatical errors should be corrected at revision, so please note any specific errors here.

Reviewer #1: Yes

Reviewer #2: Yes

**********

5. Review Comments to the Author

Please use the space provided to explain your answers to the questions above. You may also include additional comments for the author, including concerns about dual publication, research ethics, or publication ethics. (Please upload your review as an attachment if it exceeds 20,000 characters)

Reviewer #1: This is a well reasoned study on a topic of importance in bioarchaeology. The work also contributes to dental anthropology and researchers interested in growth and development. I have few substantive comments or critiques of the manuscript. The sample size could be larger, but this seems unavoidable. As the authors note, the power of the statistical analyses is diminished because of this. Still the work is worthy of publication.

The paper is clearly written and easy to follow.

The work is well researched, with an expansive bibliography.

I have only a few comments.

1) the discussion of cusp tip spacing throughout the manuscript could use some minor clarification. I think I understand what the paper is saying but consistently differentiating and referencing whether the cusp tips of the EDJ or the enamel surface would help clarify some of the confusion I had reading through this section. A figure would help greatly.

2) measuring inter-cusp distances on physical casts is not unheard of but is typically done with a labor intensive digital approach. Does observer error data suggest these date are accurate? I assume the tips were marked prior to measuring (did I miss this). If so, a statement should be added to this effect.

3) Although perhaps not needed for this study, I do wonder how visible the LEH defects (not discolorations) are on the casts. There are numerous casted populations like the one used here. Some recent studies have used LEH data collected form casts I have seen clear LEH on casts myself. I wonder how much sensitivity is lost when collecting such data. This would be a nice addition, or perhaps for a tech note elsewhere.

4) Using a breakpoint of 1 for Carabelli's trait is stated as necessary due to the low sample frequency of this trait. Is it possible to run the analyses using true cusp forms of this trait only? What are the ordinal scores for Carabelli's and cusp 5 in this sample? That would be useful information to include somewhere.

Reviewer #2: I consider the article highly valuable, as it presents new evidence on an aspect of particular importance in bioarchaeological studies. Although several studies have progressively reassessed the genetic contribution to the expression of dental morphological traits, this new contribution refines the discussion by focusing on the timing of metabolic stress events in relation to the formation process of the first permanent molar.

In my opinion, the article is well structured and clearly presented, and is therefore already suitable for publication.

I have only a few minor comments:

A. I was struck by the use of the term “adult teeth,” especially since the study focuses on a specific stage of their developmental process. I would perhaps retain the more commonly used and universally recognizable terminology, namely “permanent teeth.”

B. The citation of the work by Skinner and colleagues should be slightly reformulated. It is not Skinner et al. themselves who identify “a noticeable decrease in heritability of tooth size over the last few decades”; rather, in a study based on pigs, they discuss and report observations made by other authors. It may be sufficient to rephrase the sentence by stating that Skinner and colleagues, referring to previous studies, comment on this decrease.

C. In any case, the Skinner reference in the bibliography contains a typo error in the parentheses, and the scientific name of Sus scrofa should follow standard nomenclature: it should be in italic, with “scrofa” in lower case.

D. Finally, the bibliography should be reformatted as the final version of the article will be ready.

**********

6. PLOS authors have the option to publish the peer review history of their article (what does this mean?). If published, this will include your full peer review and any attached files.

If you choose “no”, your identity will remain anonymous but your review may still be made public.

**Do you want your identity to be public for this peer review?**  For information about this choice, including consent withdrawal, please see our Privacy Policy.

Reviewer #1: No

Reviewer #2: No

**********

[NOTE: If reviewer comments were submitted as an attachment file, they will be attached to this email and accessible via the submission site. Please log into your account, locate the manuscript record, and check for the action link "View Attachments". If this link does not appear, there are no attachment files.]

To ensure your figures meet our technical requirements, please review our figure guidelines: https://journals.plos.org/plosone/s/figures

You may also use PLOS’s free figure tool, NAAS, to help you prepare publication quality figures: https://journals.plos.org/plosone/s/figures#loc-tools-for-figure-preparation.

NAAS will assess whether your figures meet our technical requirements by comparing each figure against our figure specifications.

# Author comment: Author response to Decision Letter 1

**DOI:** 10.1371/journal.pone.0354698.r002

22 Jun 2026

Ajaya Bhattarai, Ph.D.

Academic Editor

PLOS One

Dear Dr. Bhattarai and PLOS One Manuscript Editors and Reviewers,

My colleagues and I are pleased to submit our revised manuscript titled Tracing early life stress in human molar morphology: Associations between linear enamel hypoplasia and maxillary first molar form. We thank you, the Academic Editor, and our reviewers for your thoughtful and insightful comments.

Most of the comments we received related to clarifying aspects of the methods and results, and editing citations. These comments were highly valuable to help clarify important points within this manuscript. Below please find our responses to the comments made by the Academic Editor and each reviewer.

Academic Editor:

Comment 1: Please ensure that your manuscript meets PLOS ONE's style requirements, including those for file naming.

Response: The manuscript and file names are in accordance with PLOS ONE’s style requirements.

Comment 2: In your manuscript, please provide additional information regarding the specimens used in your study. Ensure that you have reported human remain specimen numbers and complete repository information, including museum name and geographic location.

Response: A supplemental table (new S1 Table) was added with participation identification numbers as well as the institution and location where the casts are housed. However, after resubmission, we received an email saying that all human subject information should be de-identified. Because there is no other subject identification, this table was removed from the supporting information. We changed lines 316-317 to state where the casts were housed.

Comment 3: Please state what role the funders took in the study.

Response: The following amended Role of Funder statement has been added to the cover letter: The funders had no role in study design, data collection and analysis, decision to publish, or preparation of the manuscript.

Comment 4: In the online submission form you indicate that your data is not available for proprietary reasons and have provided a contact point for accessing this data. Please note that your current contact point is a co-author on this manuscript. According to our Data Policy, the contact point must not be an author on the manuscript and must be an institutional contact, ideally not an individual. Please revise your data statement to a non-author institutional point of contact, such as a data access or ethics committee, and send this to us via return email. Please also include contact information for the third party organization, and please include the full citation of where the data can be found.

Response: These data have been added to OSF with the following link: https://osf.io/zh9k8/overview?view_only=6a646d054c54462cbac5c3f5559929b7

Comment 5: Please amend the manuscript submission data (via Edit Submission) to include author Debbie Guatelli-Steinberg.

Response: Debbie Guatelli-Steinberg has been amended via Edit Submission.

Comment 6: Please amend your authorship list in your manuscript file to include author Debra Guatelli-Steinberg.

Response: Debbie Guatelli-Steinberg has been amended in the manuscript author list.

Comment 7: Please include your full ethics statement in the ‘Methods’ section of your manuscript file. In your statement, please include the full name of the IRB or ethics committee who approved or waived your study, as well as whether or not you obtained informed written or verbal consent. If consent was waived for your study, please include this information in your statement as well.

Response: Lines 305-307 have been updated as follows: This portion of the study was approved by the Mexican National Institute of Nutrition and Hampshire College’s Institutional Review Boards (IRB) [63]. Informed written consent was obtained prior to participation in the study.

Comment 8: If the reviewer comments include a recommendation to cite specific previously published works, please review and evaluate these publications to determine whether they are relevant and should be cited. There is no requirement to cite these works unless the editor has indicated otherwise.

Response: Thank you for this comment. The suggestion from Reviewer 2 was helpful in clarifying that the statement was made based on a different study. Therefore, lines 55-56 have been changed to accurately reflect what the cited article presented.

Comment 9: Please review your reference list to ensure that it is complete and correct. If you have cited papers that have been retracted, please include the rationale for doing so in the manuscript text, or remove these references and replace them with relevant current references. Any changes to the reference list should be mentioned in the rebuttal letter that accompanies your revised manuscript. If you need to cite a retracted article, indicate the article’s retracted status in the References list and also include a citation and full reference for the retraction notice.

Response: All citations have been updated to reflect expectations of the journal.

Reviewer 1:

Comment 1: the discussion of cusp tip spacing throughout the manuscript could use some minor clarification. I think I understand what the paper is saying but consistently differentiating and referencing whether the cusp tips of the EDJ or the enamel surface would help clarify some of the confusion I had reading through this section. A figure would help greatly.

Response: Thank you for your comment. Lines 173-174, 221, and 608 have been changed to clarify this point. Fig 3 is meant to demonstrate the locations of each intercusp distance measurement. To help clarify this, “of enamel cusp tips” has been added to line 387 to communicate that we measured cusp spacing using enamel cusp tips rather than from the EDJ.

Comment 2: measuring inter-cusp distances on physical casts is not unheard of but is typically done with a labor intensive digital approach. Does observer error data suggest these date are accurate? I assume the tips were marked prior to measuring (did I miss this). If so, a statement should be added to this effect.

Response: Thank you for allowing us to clarify. Lines 322-324 were added with two citations showing there are no statistically significant differences between cast and image measurements. Because this added two citations, numbering was updated throughout the rest of the manuscript and in the references cited section. Line 382 was altered to show that cusp tips were marked prior to measurement.

Comment 3: Although perhaps not needed for this study, I do wonder how visible the LEH defects (not discolorations) are on the casts. There are numerous casted populations like the one used here. Some recent studies have used LEH data collected form casts I have seen clear LEH on casts myself. I wonder how much sensitivity is lost when collecting such data. This would be a nice addition, or perhaps for a tech note elsewhere.

Response: Thank you for your comment and allowing us to clarify within the manuscript. LEH were recorded from the actual dentition, not the casts, by AHG. Lines 328-329 have been changed to clarify the source of these data.

Comment 4: Using a breakpoint of 1 for Carabelli's trait is stated as necessary due to the low sample frequency of this trait. Is it possible to run the analyses using true cusp forms of this trait only? What are the ordinal scores for Carabelli's and cusp 5 in this sample? That would be useful information to include somewhere.

Response: Thank you for this suggestion. A figure (new fig 5) has been added, showing the ordinal breakdown of trait expression for individuals with and without LEH for each Carabelli and C5 traits.

Reviewer 2:

Comment 1: I was struck by the use of the term “adult teeth,” especially since the study focuses on a specific stage of their developmental process. I would perhaps retain the more commonly used and universally recognizable terminology, namely “permanent teeth.”

Response: Thank you for your comment. Lines 29, 34, 51 and 60 have been changed from “adult” to “permanent”.

Comment 2: The citation of the work by Skinner and colleagues should be slightly reformulated. It is not Skinner et al. themselves who identify “a noticeable decrease in heritability of tooth size over the last few decades”; rather, in a study based on pigs, they discuss and report observations made by other authors. It may be sufficient to rephrase the sentence by stating that Skinner and colleagues, referring to previous studies, comment on this decrease.

Response: Thank you very much for pointing this out. Lines 55-56 have been changed to reflect the fact that Skinner and colleagues are basing this statement based on patterns observed from previous studies.

Comment 3: In any case, the Skinner reference in the bibliography contains a typo error in the parentheses, and the scientific name of Sus scrofa should follow standard nomenclature: it should be in italic, with “scrofa” in lower case.

Response: Thank you for your comment. Lines 687-689 have been changed.

Comment 4: Finally, the bibliography should be reformatted as the final version of the article will be ready.

Response: All citations have been reformatted to match expectations of the journal.

Thank you for consideration of our revised manuscript and for the opportunity to revise our submitted version.

Sincerely,

Erin C. Blankenship-Sefczek, Ph.D., Alan H. Goodman Ph.D., Mark Hubbe Ph.D., John P. Hunter Ph.D., Timothy M. Sefczek Ph.D., and Debbie Guatelli-Steinberg Ph.D.

Attachment Submitted filename: Response to Reviewers.docx

# Editor report: Decision Letter 1

**DOI:** 10.1371/journal.pone.0354698.r003

**Authors:** Ajaya Bhattarai

12 Jul 2026

Tracing early life stress in human molar morphology: Associations between linear enamel hypoplasia and maxillary first molar form

PONE-D-26-21105R1

Dear Dr. Blankenship-Sefczek,

We’re pleased to inform you that your manuscript has been judged scientifically suitable for publication and will be formally accepted for publication once it meets all outstanding technical requirements.

Within one week, you’ll receive an e-mail detailing the required amendments. When these have been addressed, you’ll receive a formal acceptance letter and your manuscript will be scheduled for publication.

An invoice will be generated when your article is formally accepted. Please note, if your institution has a publishing partnership with PLOS and your article meets the relevant criteria, all or part of your publication costs will be covered. Please make sure your user information is up-to-date by logging into Editorial Manager at Editorial Manager® and clicking the ‘Update My Information' link at the top of the page. For questions related to billing, please contact billing support.

If your institution or institutions have a press office, please notify them about your upcoming paper to help maximize its impact. If they’ll be preparing press materials, please inform our press team as soon as possible -- no later than 48 hours after receiving the formal acceptance. Your manuscript will remain under strict press embargo until 2 pm Eastern Time on the date of publication. For more information, please contact onepress@plos.org.

Kind regards,

Ajaya Bhattarai

Academic Editor

PLOS One

Additional Editor Comments (optional):

The revised manuscript looks fine.

Reviewers' comments:

# Editor report: Acceptance letter

**DOI:** 10.1371/journal.pone.0354698.r004

**Authors:** Ajaya Bhattarai

PONE-D-26-21105R1

PLOS One

Dear Dr. Blankenship-Sefczek,

I'm pleased to inform you that your manuscript has been deemed suitable for publication in PLOS One. Congratulations! Your manuscript is now being handed over to our production team.

At this stage, our production department will prepare your paper for publication. This includes ensuring the following:

* All references, tables, and figures are properly cited

* All relevant supporting information is included in the manuscript submission,

* There are no issues that prevent the paper from being properly typeset

You will receive further instructions from the production team, including instructions on how to review your proof when it is ready. Please keep in mind that we are working through a large volume of accepted articles, so please give us a few days to review your paper and let you know the next and final steps.

Lastly, if your institution or institutions have a press office, please let them know about your upcoming paper now to help maximize its impact. If they'll be preparing press materials, please inform our press team within the next 48 hours. Your manuscript will remain under strict press embargo until 2 pm Eastern Time on the date of publication. For more information, please contact onepress@plos.org.

You will receive an invoice from PLOS for your publication fee after your manuscript has reached the completed accept phase. If you receive an email requesting payment before acceptance or for any other service, this may be a phishing scheme. Learn how to identify phishing emails and protect your accounts at https://explore.plos.org/phishing.

If we can help with anything else, please email us at customercare@plos.org.

Thank you for submitting your work to PLOS ONE and supporting open access.

Kind regards,

PLOS ONE Editorial Office Staff

on behalf of

Dr. Ajaya Bhattarai

Academic Editor

PLOS One
